# Uptake, effectiveness and safety of COVID-19 vaccines in individuals at clinical risk due to immunosuppressive drug therapy or transplantation procedures: a population-based cohort study in England

**DOI:** 10.1186/s12916-024-03457-1

**Published:** 2024-06-10

**Authors:** Daniel Tzu-Hsuan Chen, Emma Copland, Jennifer A. Hirst, Emma Mi, Sharon Dixon, Carol Coupland, Julia Hippisley-Cox

**Affiliations:** 1 Nuffield Department of Primary Care Health Science, University of Oxford, Radcliffe Observatory Quarter, Woodstock Road, Oxford, OX2 6GG UK; 2 Centre for Academic Primary Care, School of Medicine, University of Nottingham, Nottingham, NG7 2UH UK

**Keywords:** Population-based, Immunocompromised, COVID-19, COVID-19 vaccination, Vaccine effectiveness, Vaccine safety, Vaccine uptake

## Abstract

**Background:**

Immunocompromised individuals are at increased risk of severe COVID-19 outcomes, underscoring the importance of COVID-19 vaccination in this population. The lack of comprehensive real-world data on vaccine uptake, effectiveness and safety in these individuals presents a critical knowledge gap, highlighting the urgency to better understand and address the unique challenges faced by immunocompromised individuals in the context of COVID-19 vaccination.

**Methods:**

We analysed data from 12,274,946 people in the UK aged > 12 years from 01/12/2020 to 11/04/2022. Of these, 583,541 (4.8%) were immunocompromised due to immunosuppressive drugs, organ transplants, dialysis or chemotherapy. We undertook a cohort analysis to determine COVID-19 vaccine uptake, nested case–control analyses adjusted for comorbidities and sociodemographic characteristics to determine effectiveness of vaccination against COVID-19 hospitalisation, ICU admission and death, and a self-controlled case series assessing vaccine safety for pre-specified adverse events of interest.

**Results:**

Overall, 93.7% of immunocompromised individuals received at least one COVID-19 vaccine dose, with 80.4% having received three or more doses. Uptake reduced with increasing deprivation (hazard ratio [HR] 0.78 [95%CI 0.77–0.79] in the most deprived quintile compared to the least deprived quintile for the first dose). Estimated vaccine effectiveness against COVID-19 hospitalisation 2–6 weeks after the second and third doses compared to unvaccinated was 78% (95%CI 72–83) and 91% (95%CI 88–93) in the immunocompromised population, versus 85% (95%CI 83–86) and 86% (95%CI 85–89), respectively, for the general population. Results showed COVID-19 vaccines were protective against intensive care unit (ICU) admission and death in both populations, with effectiveness of over 92% against COVID-19-related death and up to 95% in reducing ICU admissions for both populations following the third dose. COVID-19 vaccines were generally safe for immunocompromised individuals, though specific doses of ChAdOx1, mRNA-1273 and BNT162b2 raised risks of specific cardiovascular/neurological conditions.

**Conclusions:**

COVID-19 vaccine uptake is high in immunocompromised individuals on immunosuppressive drug therapy or who have undergone transplantation procedures, with documented disparities by deprivation. Findings suggest that COVID-19 vaccines are protective against severe COVID-19 outcomes in this vulnerable population, and show a similar safety profile in immunocompromised individuals and the general population, despite some increased risk of adverse events. These results underscore the importance of ongoing vaccination prioritisation for this clinically at-risk population to maximise protection against severe COVID-19 outcomes.

**Supplementary Information:**

The online version contains supplementary material available at 10.1186/s12916-024-03457-1.

## Background

There are over 500,000 people in England who are immunocompromised, with the vast majority due to immune-suppressive drug treatment or organ transplant procedures [1]. Suppression of the immune system has been considered a potentially major risk factor for severe outcomes from infectious diseases like COVID-19. Throughout the COVID-19 pandemic, immunocompromised people were more likely to be hospitalised, admitted to an intensive care unit (ICU) or die from SARS-CoV-2 infection compared to people without weakened immune systems [2–4]. Notably, COVID-19 mortality rates have not declined for immunocompromised people at the same rate as for their immunocompetent peers over time [5].

Vaccination remains an important preventive measure against COVID-19. The UK started its COVID-19 vaccination programme in December 2020, prioritising clinically vulnerable groups, including those who were immunocompromised [6]. By May 2023, the UK Joint Committee on Vaccination and Immunisation (JCVI) recommended a booster dose in autumn 2023 for those aged 6 months and over who are immunosuppressed, marking the seventh dose for many within this group [7]. While the general trend indicates a high COVID-19 vaccination uptake across the UK, there have been concerning reports of socioeconomic and racial disparities in vaccine acceptance, especially within clinically vulnerable cohorts [8]. Despite the emphasis on vaccination, population-wide evidence on COVID-19 vaccine uptake among immunocompromised individuals remains limited.

Though concerted efforts have been made globally to prioritise the vaccination of clinically vulnerable people, population-based evidence on the effectiveness of the vaccines, especially among the immunocompromised, remains scarce. Evidence from hospital cohorts suggests that immunocompromised people have a poorer immune response to COVID-19 vaccination, characterised by lower seroconversion rates and diminished antibody titres post-vaccination compared to immunocompetent individuals [9, 10]. Further studies encompassing people on immunosuppressive therapy, those undergoing haemodialysis, stem cell transplant recipients and cancer patients [11, 12] have shown that third and fourth vaccine doses may enhance both humoral and T-cell immunity, and be effective against the Omicron variant in various immunocompromised subgroups. However, immune responses vary between immunocompromising conditions and evidence suggests a faster waning of immunity over time for some patients [13–15]. The clinical implications of these immune responses remain unclear, with vaccine effectiveness reports showing a wide range from 32 to 83% against COVID-19-related hospitalisation and 62 to 95% against COVID-19-related death following third and fourth doses in immunocompromised populations [16–19]. Notably, most of these studies have focused on specific patient groups, such as those receiving haemodialysis or those diagnosed with cancer [20, 21].

The safety profile of COVID-19 vaccines in these populations also remains inconclusive. Although COVID-19 vaccination has been shown to be safe in the general population, there are emerging concerns about potential immune-related adverse events in specific patient groups. Some studies have indicated associations between COVID-19 vaccines with some immune-related adverse events, including the exacerbation of pre-existing conditions [22–24]. However, only very few small studies have been conducted, and population-based studies on immunocompromised people remain limited in this regard [25–27]. There is preliminary evidence suggesting vaccination might be linked to a relapse of autoimmune rheumatic disease post-vaccination, but conversely, other studies found no increased disease activity in those with autoimmune conditions post-vaccination, and any autoimmune sequelae that did arise were mild and had good outcomes [28, 29].

Given the ongoing vaccination of clinically vulnerable groups in the UK, it is crucial to obtain a comprehensive understanding of vaccine uptake, particularly by ethnicity and socioeconomic status, in the immunocompromised population. Moreover, there is urgent need to assess the effectiveness and safety of COVID-19 vaccines in this clinically at-risk group. In light of this, the present work reports findings from a large population-based study using real-word data to evaluate COVID-19 vaccine uptake, effectiveness and safety in individuals likely to have compromised immune systems after receiving immunosuppressive drug therapy or having undergone transplantation procedures. These findings will inform health care policy and guide current and future COVID-19 vaccination programmes for these clinically at-risk groups.

## Methods

### Data sources and settings

We used the QResearch primary care database, an anonymised research database of patients from approximately 1500 general practices in England covering a representative sample of around 20% of the English population [30]. The database was linked to Hospital Episode Statistics (HES) data (NHS England); National Cancer Registration and Analysis Service data (NCRAS, NHS England); civil registration national data for mortality with date and causes of death (Office for National Statistics [ONS]); Second Generation Surveillance System (SGSS) for SARS-CoV-2 testing data and National Immunisation Database (NIMS) of COVID-19 vaccinations, to identify data on vaccine doses, dates and types of vaccine (mainly, ChAdOx-nCov19 [or ChAdOx1, AstraZeneca/Oxford], BNT162b2 [Pfizer–BioNTech] and mRNA1273 [Moderna]).

### Study population and identification of immunocompromised individuals

In line with the UK’s COVID-19 vaccination policies during majority of the study period, we included a fixed cohort of individuals aged 12 years and over at study entry, who were eligible for vaccination and were registered with a general practice contributing data to the QResearch primary care database on 1st December 2020. The cohort was followed up until 11th April 2022 (last data update at time of analysis). The study age criterion reflects eligibility for vaccination at the point of data entry into our study. Guided by the literature [31] and clinical experts, we identified an immunocompromised population in the QResearch database with a diagnostic or drug prescription code in their medical record for one or more immune-modifying drugs, including oral steroids (specified in British National Formulary (BNF) chapter 8.2) or chemotherapy in the 6 months prior to the study start date and those who had received a solid organ transplant, dialysis or bone marrow transplant within the 24 months preceding the study start date. Conditions related to HIV (human immunodeficiency virus) alone were not included in this study. Individuals who met the above inclusion criteria were included in the following four non-mutually exclusive subgroups: (i) organ transplant procedures (solid organ or bone marrow), (ii) renal transplant or dialysis, (iii) receiving immune-modifying drugs or (iv) receiving chemotherapy, and are referred to collectively as “the immunocompromised population”. The remaining individuals in the dataset are referred to as the general population. The full list of criteria to be identified as immunocompromised is described in Additional file 1.

### Objectives and general analysis

Following pre-specified analysis plans [Additional files 3–5 [32–39], our study had three objectives: (i) to describe the characteristics of the populations by subgroups and report vaccine uptake, (ii) to estimate effectiveness of COVID-19 vaccines during the study period and (iii) to estimate risks of safety outcomes for COVID-19 vaccines. The study included analysis of vaccine uptake data from the first through fourth doses and analysed effectiveness and safety for the first three doses. Covariates for participants who entered the study population were collected at their time of entry in December 2020, ensuring consistency in data analysis.

Descriptive demographics to report age, sex, body mass index (BMI), ethnic group and Townsend quintile of deprivation were tabulated as mean and standard deviation (SD) or numbers and percentage for the immunocompromised population, subgroups of immunocompromised people and in the general population.

**Fig. 1:**
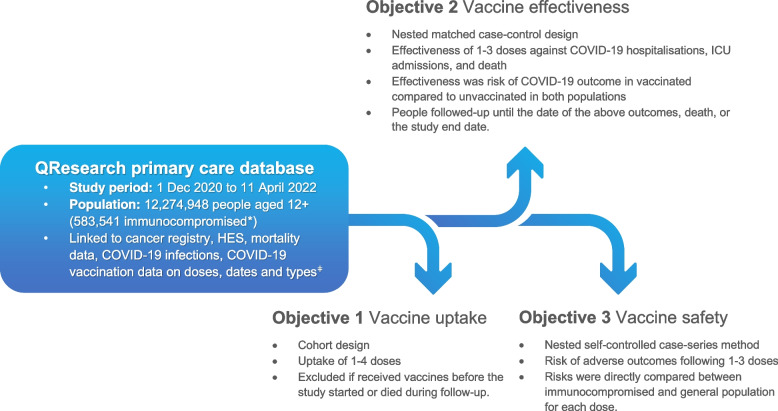
Diagram of the data sources, study methods and objectives. Note: * immunocompromised due to immunosuppressive drugs, organ transplantation, undergoing dialysis or receiving chemotherapy; ǂ mainly, ChAdOx1 [AstraZeneca/Oxford], BNT162b2 [Pfizer–BioNTech] and mRNA1273 [Moderna]

Figure 1 illustrates the study methods and designs for the three study objectives, which are described separately as follows. All analyses were performed using Stata 17MP (StataCorp, TX).

#### Vaccine uptake

##### Outcomes and study design

We reported uptake of one to four COVID-19 vaccine doses during the study period. A cohort study design was used, with the study period from 1st December 2020 (date of first vaccination in England) to 11th April 2022, the latest date for which linked data were available. Individuals who received vaccines before the study started or died during follow-up were excluded. For the analyses of uptake of each dose, participants entered the study on the dates when each dose was made available to clinically vulnerable groups in the UK: 1st December 2020, 1st March 2021, 14th September 2021 and 1st January 2022, for each of the four doses, respectively, and people were censored on the earliest of date of vaccination or the study end date.

##### Statistical analysis

Results were presented as numbers and percentage receiving one, two, three and four COVID-19 vaccine doses in the immunocompromised group and general population using descriptive tables and time to uptake of each dose. Kaplan–Meier curves were used to compare time to uptake of each vaccine dose (first to fourth) between the immunocompromised group and the general population.

An additional aim of the vaccine uptake analysis was to identify sociodemographic factors associated with uptake of each COVID-19 vaccine dose within the immunocompromised population. This was carried out using Cox proportional hazards models adjusting for age, sex, BMI, region of England and comorbidities (identified using the QCOVID algorithm) [32]. Because over 35% of the population had missing data for BMI, multiple imputation using chained equations (MICE) was used to impute missing data. Incomplete data were imputed 10 times and the estimates were combined using Rubin’s rules [40]. We created a “missing” category for those whose ethnicity data was not recorded to ensure all eligible people were included.

#### Vaccine effectiveness

##### Outcomes and study design

We reported the occurrence of three primary outcomes to investigate COVID-19 vaccine effectiveness: COVID-19-related hospitalisation, ICU admission or death, in those who received one, two or three doses of COVID-19 vaccine compared with those who did not receive the vaccine. COVID-19-related hospitalisation was defined as a positive SARS-CoV-2 test (confirmed by reverse transcription-polymerase chain reaction) within 14 days before a hospital admission or before discharge, or hospital admission with ICD10 code for COVID-19 disease (U071) or suspected COVID-19 disease (U072). COVID-19-related ICU admission was defined as an ICU admission during a COVID-19-related hospitalisation. COVID-19-related death was defined as COVID-19 being recorded on the death certificate or death from any cause within 28 days of a laboratory-confirmed SARS-CoV-2 infection, derived from the ONS mortality register [36]. The population was followed up from the start of the study (1st December 2020) to the earliest of either the end of the study period (11th April 2022), the date of the outcome of interest or when they died.

A nested matched case–control design was used to evaluate vaccine effectiveness [35] for all three outcomes in the immunocompromised population and separately in the general population. We used incidence density matching with replacement to match each case randomly by age, sex and calendar date with up to ten controls who had not experienced the outcome of interest by that date. Vaccine effectiveness was estimated in the two populations by comparing the odds of vaccination in cases and controls for each outcome.

##### Statistical analysis

We used conditional logistic regression models to estimate odds ratios (OR) with 95% confidence intervals (95%CI) for each of the three COVID-19 outcomes following a first, second or third dose of COVID-19 vaccine compared to controls (unvaccinated individuals) in the matched case–control datasets of both populations. Models were adjusted for comorbidities (identified using the QCOVID risk algorithm) [32], previous SARS-CoV-2 infection [37] and sociodemographic characteristics (full list defined in Additional file 4). Missing values for BMI were imputed using multiple imputation and for ethnicity were categorised as missing (described above). To facilitate interpretation, (adjusted) vaccine effectiveness was estimated by 100 × (1 − OR), using the OR as an approximation of the risk ratio, and reported for both the immunocompromised and general population.

We presented vaccine effectiveness estimates from day 0 onwards to provide a comprehensive overview of the data. However, we focus on estimates 14 days or more after vaccination, as this timeframe allows for a detectable vaccine-mediated immune response to develop [34]. We considered the following exposure periods as previously reported in the literature [32, 35]: 0–13, 14–27, 28–41 and 42 + days following first dose; 0–13, 14–41, 42–97, 98–153, 154–181, 182–272 and 273 + days following second dose; and 0–13, 14–41 and 42–97 days following third dose.

Where there were sufficient data, additional analyses were performed to estimate vaccine effectiveness separately by vaccine type (ChAdOx1, BNT162b2, mRNA-1273), subgroups of immunocompromised conditions and COVID-19 variants (Alpha, Delta and Omicron BA.1) by stratifying by calendar time periods coinciding with changes in the dominance of SARS-CoV-2 variants as reported by the ONS [36] (Alpha: 18th December 2020–17th May 2021; Delta: 18th May 2021–19th December 2021; Omicron BA.1: 20th December 2021–1st March 2022).

### Vaccine safety

#### Outcomes and study design

We pre-specified 56 outcomes that were adverse events of special interest for vaccine safety as defined by the Brighton Collaboration [41], as well as outcomes identified in the emerging scientific literature, including rheumatological conditions, liver disease, blood disorders, neuroinflammatory disorders, cardiovascular disease, inflammatory skin conditions, other autoimmune disorders and allergy-related diseases (full list defined in Additional file 2).

Vaccine safety was assessed using a self-controlled case series design [38, 39], in which individuals act as their own controls. This implicitly controls for confounders that do not vary over time during the observation period. In this study design, each outcome is analysed separately, and only people who had the outcome of interest and were vaccinated with at least one dose of COVID-19 vaccine or had a positive SARS-CoV-2 test recorded during the study period were included in the analysis.

We considered the 1–28 days after first, second or third dose of COVID-19 vaccine as the exposure periods of interest for vaccine safety [23]. The 1–28 days before each dose was given were classified as separate exposure periods (“pre-risk periods”) to account for the potential bias that could be introduced by people with a recent hospital admission delaying vaccination. The day of each vaccination was also included as a separate exposure period and the remaining observation time was defined as the baseline period, which was used as the comparator to the exposure risk periods. The primary exposure was any COVID-19 vaccine, but we also investigated differences in the safety profiles for each vaccine type: ChAdOx1, BNT162b2 and mRNA-1273. We included SARS-CoV-2 infection as a separate exposure to control for the potential effect of COVID-19 infection on the risk of the safety outcomes, and differentiated between infections occurring before and after the first vaccine dose.

#### Statistical analysis

We only analysed adverse events for which at least five immunocompromised individuals experienced the outcome in the exposure period. We used Poisson regression models to generate incidence rate ratios (IRRs) of each adverse event occurring in the vaccine exposure risk period (1–28 days after first, second or third dose) compared to baseline in immunocompromised people. As a secondary analysis, we included an interaction term between the exposure and immunocompromised status to estimate the relative IRR of each outcome in immunocompromised people compared to the general population. We accounted for underlying seasonal effects by adjusting for observation time split into 2-week periods and included an offset term for length of exposure. We assessed significance at the 1% level and presented 99%CIs to account for multiple testing. We adjusted *p* values using the Bonferroni correction to further account for multiple comparisons.

We conducted sensitivity analyses to check the robustness of the results. We fitted models excluding people with a positive SARS-CoV-2 test to assess the risk of safety outcomes from the vaccine alone. We also fitted models excluding people who died during the study period as these are likely to be the most vulnerable people, and excluding people with a record of the adverse event recorded in the month prior (1st–30th November 2020) or 2 years prior (1st December 2018–30th November 2020) to the study start date as they may be more likely to experience the outcome of interest than people with no history of the outcome. We additionally tested the robustness of our results for outcomes that increase the probability of death by starting the observation period at the day of the first, second and third doses, and by fitting models without censoring for deaths due to the outcome. The sensitivity analyses are described in more detail in Additional file 5.

## Results

### Study population

Demographic characteristics of the study population are described in Table 1. The full QResearch cohort included 12,274,948 people, of whom 583,541 (4.8%) were identified as immunocompromised due to immunosuppressive drug therapy or transplantation procedures. Among this population, 546,173 (93.6%) were receiving immune-modifying drugs, 10,842 (1.9%) had received an organ transplant (solid organ, liver or bone marrow), 9926 (1.7%) had renal transplant or dialysis and 64,601 (11.1%) had chemotherapy treatments. A diagram of the subgroups included in the analysis is shown in Additional file 1. The mean age of the immunocompromised population was 61.3 years (SD 17.9) and for the general population was 43.2 years (19.6).

**Table 1:** Demographic characteristics, COVID-19 vaccination status and COVID-19-related outcomes of the study population (*N* = 12,274,948): general population (*N* = 11,691,407) and immunocompromised population* (*N* = 583,541). Figures are column (%) unless otherwise specified

	Study population (*N*=12,274,948)		Immunocompromised subgroups* (*N* = 583,541)			
	**General population**	**Immunocompromised**	**Transplant**	**Dialysis or renal transplant**	**Immune-modifying drugs**	**Chemotherapy**
**Demographic characteristics**						
Total (*N*)	11,691,407	583,541	10,842	9926	546,173	64,601
Age, years mean (SD)	43.2 (19.6)	61.3 (17.9)	52.5 (16.3)	60.6 (16.8)	61.3 (18.0)	66.9 (15.1)
Female sex (%)	5,841,993 (50.0)	293,266 (50.3)	4471 (41.2)	4023 (40.6)	273,389 (50.1)	33,084 (51.2)
BMI, kg/m^2^ mean (SD)	26.5 (5.7)	28.5 (6.2)	27.0 (5.5)	28.0 (6.2)	28.6 (6.2)	26.8 (5.2)
Townsend quintile of deprivation (%)						
Q1 (most affluent)	2,700,122 (23.1)	165,305 (28.3)	2601 (24.0)	1936 (19.5)	154,670 (28.3)	24,494 (37.9)
Q2	2,465,166 (21.1)	139,862 (24.0)	2476 (22.8)	1855 (18.7)	131,164 (24.0)	17,092 (26.5)
Q3	2,264,271 (19.4)	115,065 (19.7)	2065 (19.0)	2042 (20.6)	108,059 (19.8)	10,861 (16.8)
Q4	2,090,322 (17.9)	92,504 (15.9)	1870 (17.2)	2017 (20.3)	86,740 (15.9)	6979 (10.8)
Q5 (most deprived)	2,028,725 (17.4)	66,218 (11.3)	1715 (15.8)	1995 (20.1)	61,242 (11.2)	4713 (7.3)
Ethnicity (%)						
White	7,278,469 (62.3)	447,121 (76.6)	6951 (64.1)	5843 (58.9)	420,767 (77.0)	51,099 (79.1)
Indian	360,120 (3.1)	12,215 (2.1)	383 (3.5)	375 (3.8)	11,391 (2.1)	634 (1.0)
Pakistani	239,707 (2.1)	9794 (1.7)	337 (3.1)	364 (3.7)	9153 (1.7)	407 (0.6)
Chinese	140,562 (1.2)	1558 (0.3)	59 (0.5)	51 (0.5)	1395 (0.3)	135 (0.2)
Bangladeshi	158,428 (1.4)	4759 (0.8)	154 (1.4)	220 (2.2)	4368 (0.8)	231 (0.4)
Other Asian	240,852 (2.1)	6708 (1.1)	226 (2.1)	226 (2.3)	6189 (1.1)	388 (0.6)
Black Caribbean	122,032 (1.0)	5277 (0.9)	237 (2.2)	375 (3.8)	4549 (0.8)	482 (0.7)
Black African	325,306 (2.8)	6132 (1.1)	412 (3.8)	505 (5.1)	5173 (0.9)	618 (1.0)
Other	523,848 (4.5)	11,383 (2.0)	415 (3.8)	462 (4.7)	10,266 (1.9)	958 (1.5)
Not recorded	2,302,029 (19.7)	78,595 (13.5)	1668 (15.4)	1505 (15.2)	72,922 (13.4)	9649 (14.9)
**COVID-19 vaccination status**						
Unvaccinated	2,443,407 (20.9)	36,656 (6.3)	919 (8.5)	1228 (12.4)	33,136 (6.1)	3718 (5.8)
Vaccinated (≥ 1 dose)	9,248,000 (79.1)	546,885 (93.7)	9923 (91.5)	8698 (87.6)	21,037 (93.9)	60,883 (94.2)
Vaccine dose 1						
ChAdOx1	4,184,117 (35.8)	304,587 (52.2)	5345 (49.3)	4803 (48.4)	286,117 (52.4)	32,151 (49.8)
BNT162b2	4,696,834 (40.2)	239,030 (41.0)	4542 (41.9)	3866 (38.9)	223,703 (41.0)	28,614 (44.3)
mRNA-1273	365,405 (3.1)	3242 (0.6)	36 (0.3)	29 (0.3)	3132 (0.6)	113 (0.2)
Vaccine dose 2						
ChAdOx1	4,074,523 (34.9)	295,677 (50.7)	5157 (47.7)	4524 (45.6)	278,260 (50.9)	30,932 (47.9)
BNT162b2	4,305,607 (36.8)	232,765 (39.9)	4440 (41.0)	3696 (37.2)	218,039 (39.9)	27,882 (43.2)
mRNA-1273	333,107 (2.8)	3175 (0.5)	39 (0.4)	28 (0.3)	3072 (0.6)	111 (0.2)
Vaccine dose 3						
ChAdOx1	15,103 (0.1)	2289 (0.4)	61 (0.6)	43 (0.4)	2142 (0.4)	217 (0.3)
BNT162b2	5,131,289 (43.9)	414,077 (71.0)	7884 (72.7)	6257 (63)	388,912 (71.2)	48,638 (75.3)
mRNA-1273	1,507,783 (12.9)	52,428 (9.0)	550 (5.1)	468 (4.7)	50,659 (9.3)	3693 (5.7)
Vaccine dose 4						
ChAdOx1	370 (0)	103 (0)	< 5 (0)	< 5 (0)	97 (0)	< 5 (0)
BNT162b2	194,087 (1.7)	71,564 (12.3)	3687 (34)	1636 (16.5)	62,868 (11.5)	13,898 (21.5)
mRNA-1273	125,504 (1.1)	26,607 (4.6)	616 (5.7)	362 (3.6)	24,721 (4.5)	4618 (7.1)
**COVID-19 outcomes during follow-up**						
Hospitalisations	76,734 (0.7)	17,817 (3.1)	811 (7.5)	1108 (11.2)	15,964 (2.9)	1757 (2.7)
Death	17,197 (0.2)	5478 (0.9)	193 (1.8)	298 (3)	4860 (0.9)	749 (1.2)
ICU admission	6925 (0.1)	1249 (0.2)	78 (0.7)	131 (1.3)	1096 (0.2)	63 (0.1)

Note: ChAdOx1 (AstraZeneca/Oxford), BNT162b2 (Pfizer) and mRNA-1273 (Moderna), Janssen and Valneva are omitted for less than 0.01% in all populations, *ICU* Intensive care unit

* immunocompromised due to immunosuppressive drugs, organ transplantation, undergoing dialysis or receiving chemotherapy

### Vaccine uptake

Between 1st December 2020 and 11th April 2022, the immunocompromised population had higher overall vaccine coverage, with 93.7% receiving at least one dose of COVID-19 vaccine, compared to 79.1% of the general population. This elevated vaccine uptake among the immunocompromised group extended to subsequent doses as well, with 80.4% of this group receiving three or more doses, in contrast to 56.9% of the general population (Table 1). Immunocompromised people also had shorter time to vaccine uptake for all doses compared to the general population (Additional file 6: Figs. S1–S2).

**Fig. 2:**
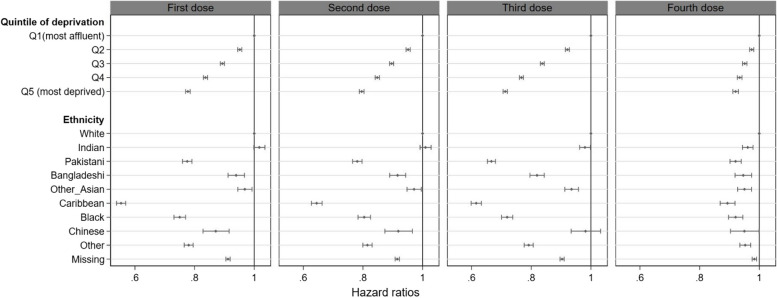
Forest plot of the multivariable Cox regression analyses of COVID-19 vaccine uptake in the immunocompromised population*. Note: * immunocompromised due to immunosuppressive drugs, organ transplantation, undergoing dialysis or receiving chemotherapy; Q1 to Q5 indicates quintiles of Townsend, from the most affluent (Q1) to the most deprived (Q5); models were adjusted for age, sex, BMI, region of England and QCOVID comorbidities

Results from multivariable Cox regression in the immunocompromised group showed a trend towards lower uptake with each increasing quintile of deprivation (Fig. 2). Immunocompromised people in the most deprived quintile were 22% (hazard ratio [HR] 0.78, 95%CI 0.77–0.79), 21% (0.79, 0.79–0.80), 29% (0.71, 0.71–0.72) and 8% (0.92, 0.91–0.93) less likely to receive a first (from 1st December 2020), second (from 1st March 2021), third (from 14th September 2021) or fourth dose (from 1st January 2022), respectively, compared to people in the most affluent quintile of the population. Compared with people of white ethnicity, uptake of all four vaccine doses was significantly lower in Pakistani, Bangladeshi, other Asian, Black Caribbean and Black African ethnicities (Fig. 2).

Subgroup analyses revealed differences in vaccine uptake by age group, dose, vaccine type, immunocompromised subgroup and demographic characteristics, as shown in Additional file 6: Figs. S3–S7.

### Vaccine effectiveness

Within the immunocompromised population, approximately 3% (*n* = 17,817) had a COVID-19-related hospital admission during follow-up, and less than 1% were admitted to the ICU (0.9%, *n* = 5478) or died (0.2%, *n* = 1249) with COVID-19. In the general population, 0.7% (*n* = 76,734) had a COVID-19-related hospital admission, 0.2% (*n* = 17,197) were admitted to the ICU and 0.1% (*n* = 6925) died with COVID-19 (Table 1).

**Fig. 3:**
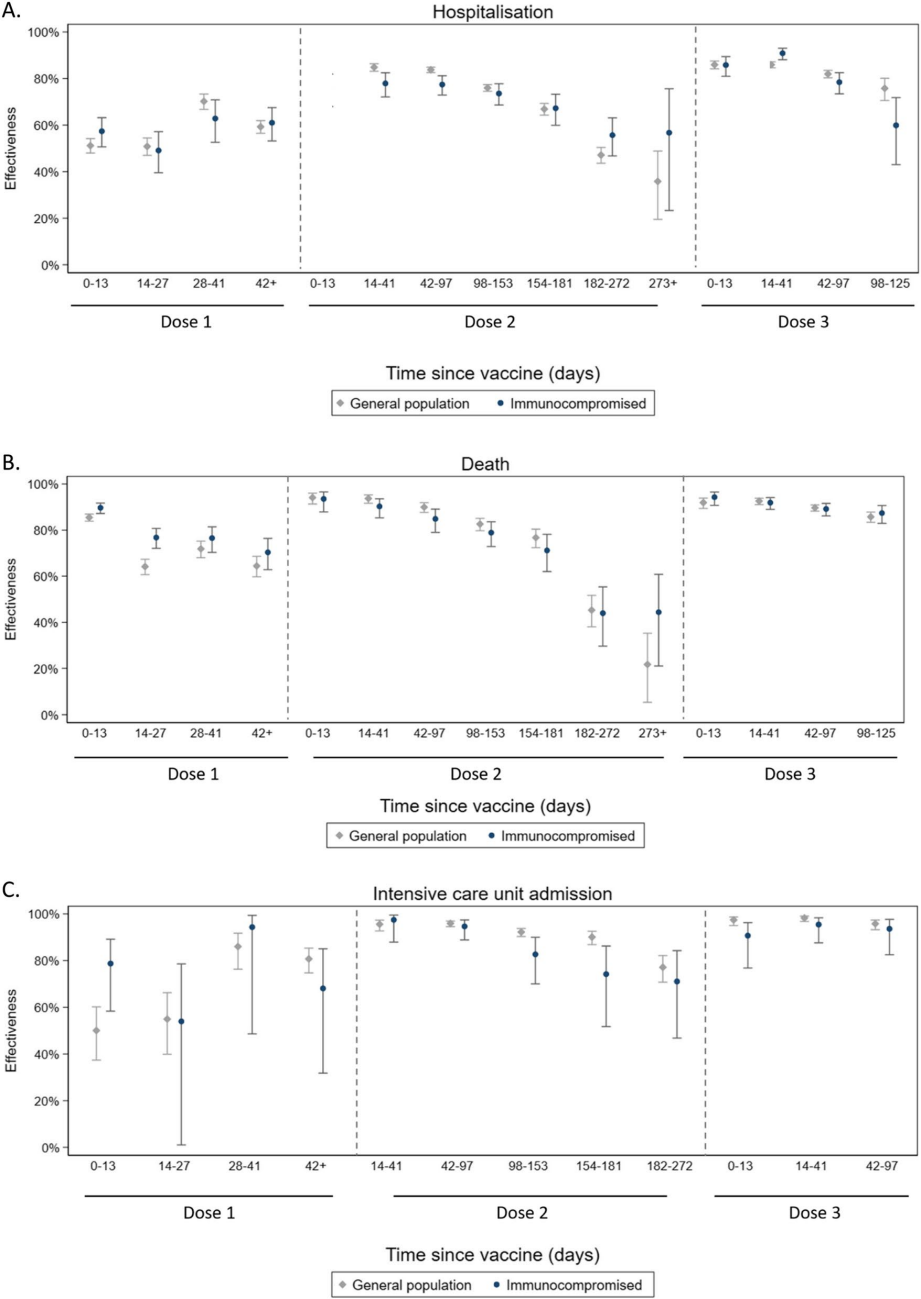
Adjusted vaccine effectiveness against COVID-19-related hospitalisation, intensive care unit (ICU) admission and death in the study population. Note: Vaccine effectiveness reported was estimated relative to the unvaccinated in both the immunocompromised and the general population; modelling was conducted separately for both populations and was further adjusted for ethnicity, Townsend, prior-COVID-19 infection, BMI, region and QCOVID comorbidities. VE: vaccine effectiveness = (1 − odds ratio)*100

In immunocompromised individuals, COVID-19 vaccination provided comparable levels of protection against COVID-19-related hospitalisation to that observed in the general population. In the 14–41 days following a second dose of COVID-19 vaccine, vaccine effectiveness compared to unvaccinated group was 78% (95%CI 72–83%) in the immunocompromised population and 85% (83–86%) in the general population (Fig. 3A, Table 2). However, following a third dose (14–41 days), vaccine effectiveness showed higher protection in immunocompromised people (91% [88–93%]) compared with the general population (86% [85–89%]). Vaccine effectiveness decreased over time in both populations to below 60% after 182 days since the second dose and to below 80% after 98 days since the third dose.

**Table 2:** Adjusted vaccine effectiveness (VE) against COVID-19-related hospitalisation, intensive care unit (ICU) admission and death in the general and the immunocompromised population using a matched case–control study design, with vaccine effectiveness (1 − OR) with 95% confidence intervals

	**COVID-19-related hospitalisation**					**COVID-19-related ICU admission**					**COVID-19-related death**				
	***N*** ** controls**	***N*** ** cases**	**VE**	**95%CI**		***N*** ** controls**	***N*** ** cases**	**VE**	**95%CI**		***N*** ** controls**	***N*** ** cases**	**VE**	**95%CI**	
**Immunocompromised population**															
** No vaccine**	20,806	3023	0	0	0	3846	462	0	0	0	20,806	3023	0	0	0
** 1st dose (days after)**															
0–13	5171	104	0.58	0.51	0.63	294	12	0.79	0.68	0.89	5171	104	0.9	0.87	0.92
14–27	3449	194	0.5	0.4	0.57	130	11	0.73	0.6	0.81	3449	194	0.77	0.72	0.81
28–41	1977	134	0.63	0.53	0.71	151	15	0.74	0.561	0.89	1977	134	0.77	0.7	0.81
42 +	1819	200	0.62	0.54	0.68	100	15	0.68	0.55	0.85	1819	200	0.7	0.63	0.76
** 2nd dose (days after)**															
0–13 days	750	11	0.76	0.68	0.82	37	< 5	–	–	–	750	11	0.94	0.88	0.97
14–41	1056	30	0.78	0.72	0.83	90	12	0.97	0.88	0.99	1056	30	0.9	0.85	0.94
42–97	1306	87	0.78	0.73	0.81	401	16	0.95	0.89	0.97	1306	87	0.85	0.79	0.89
98–153	3106	265	0.74	0.69	0.78	569	49	0.83	0.7	0.9	3106	265	0.79	0.73	0.84
154–181	1796	172	0.67	0.59	0.73	287	28	0.74	0.52	0.86	1796	172	0.71	0.62	0.78
182–272	1767	429	0.55	0.46	0.63	233	38	0.71	0.47	0.84	1767	429	0.44	0.3	0.55
273 +	236	100	0.56	0.22	0.75	8	< 5	–	–	–	236	100	0.44	0.21	0.61
** 3rd dose (days after)**															
0–13 days	951	22	0.86	0.81	0.89	156	8	0.91	0.77	0.96	951	22	0.94	0.91	0.97
14–41	2053	86	0.91	0.88	0.93	224	6	0.95	0.88	0.98	2053	86	0.92	0.89	0.94
42–97	4449	303	0.78	0.73	0.82	155	8	0.94	0.83	0.98	4449	303	0.89	0.86	0.92
98–125	1947	153	0.59	0.41	0.71	11	< 5	–	–	–	1947	153	0.87	0.83	0.91
**General population**															
** No vaccine**	73,365	10,741	0	0	0	30,660	4102	0	0	0	73,365	10,741	0	0	0
** 1st dose (days after)**															
0–13	17,715	465	0.51	0.48	0.54	1517	112	0.6	0.47	0.7	17,715	465	0.86	0.84	0.87
14–27	12,215	855	0.51	0.47	0.54	881	71	0.55	0.4	0.66	12,215	855	0.64	0.61	0.67
28–41	6698	443	0.7	0.67	0.73	522	17	0.86	0.76	0.92	6698	443	0.72	0.68	0.75
42 +	5949	642	0.59	0.56	0.62	1230	77	0.81	0.75	0.85	5949	642	0.64	0.6	0.69
** 2nd dose (days after)**															
0–13 days	2656	29	0.85	0.82	0.87	525	< 5	–	–	–	2656	29	0.94	0.91	0.96
14–41	3608	62	0.85	0.83	0.86	1136	18	0.96	0.93	0.97	3608	62	0.94	0.92	0.95
42–97	3610	155	0.84	0.83	0.85	3397	66	0.96	0.95	0.97	3610	155	0.9	0.88	0.92
98–153	6242	431	0.76	0.75	0.77	3653	147	0.92	0.9	0.94	6242	431	0.83	0.8	0.85
154–181	3704	308	0.67	0.64	0.69	1541	76	0.9	0.87	0.93	3704	308	0.77	0.72	0.8
182–272	4327	940	0.47	0.44	0.5	1022	131	0.77	0.71	0.82	4327	940	0.45	0.38	0.52
273 +	708	316	0.36	0.19	0.49	11	< 5	–	–	–	708	316	0.22	0.05	0.35
** 3rd dose (days after)**															
0–13 days	2380	68	0.86	0.84	0.88	747	10	0.97	0.95	0.99	2380	68	0.92	0.89	0.94
14–41	5121	180	0.86	0.85	0.89	1081	12	0.98	0.97	0.99	5121	180	0.93	0.91	0.94
42–97	12,547	719	0.82	0.8	0.84	869	36	0.96	0.93	0.97	12,547	719	0.9	0.88	0.91
98–125	6101	430	0.76	0.71	0.8	31	< 5	–	–	–	6101	430	0.86	0.83	0.88

Note: Vaccine effectiveness reported was estimated relative to the unvaccinated in both the immunocompromised and the general population; cases and controls were matched by age, sex, calendar date for both the immunocompromised and the general population; modelling was conducted separately for both populations and was further adjusted for ethnicity, Townsend, prior-COVID-19 infection, BMI, region and QCOVID comorbidities. VE: vaccine effectiveness = (1 − odds ratio)*100; –: no data point due to insufficient numbers of cases

COVID-19 vaccines were highly protective against COVID-19-related death in both the immunocompromised and general population. Vaccine effectiveness was 90% (85–94%) 14–41 days after a second dose and 92% (89–94%) 14–41 days after a third dose in the immunocompromised population, and 94% (92–95%) and 93% (91–94%), respectively, in the general population (Fig. 3B). Protection waned over time in both populations to below 70% after 182 days from the second dose and to below 90% after 98 days from the third dose.

Vaccine effectiveness against COVID-19-related ICU admission was highest following a second and third dose in the immunocompromised group (97% [88–99%] and 95% [88–98%] after 14–41 days, respectively). Similar levels of protection were observed in the general population (96% [93–97%] and 98% [97–99%]) (Fig. 3C). Vaccine effectiveness remained above 70% in both populations up to 272 days after the second dose.

Compared to unvaccinated people, analyses of ChAdOx1 and BNT162b2 both showed significant protection against COVID-19-related hospitalisation in the immunocompromised population following the first two doses (Additional file 6: Fig. S8). Vaccine effectiveness during the Alpha, Delta and Omicron BA.1 variant periods [36] was around 60%, 81% and 55% in the three periods against hospitalisation, respectively, among the immunocompromised population (Additional file 6: Figs. S9–S10).

### Vaccine safety in the immunocompromised population

**Fig. 4:**
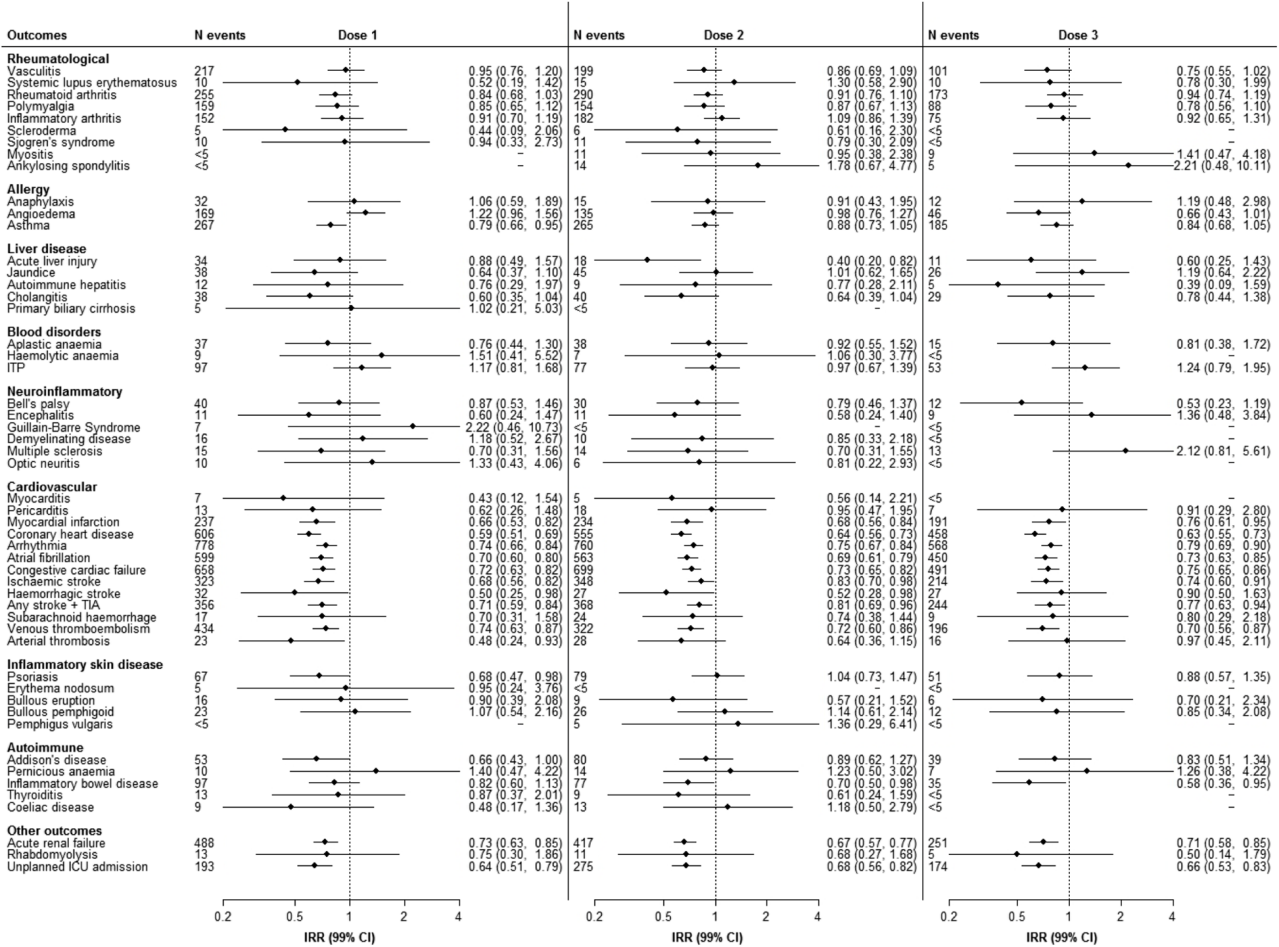
Risk of serious outcomes following any COVID-19 vaccination in immunocompromised people*. Note: * immunocompromised due to immunosuppressive drugs, organ transplantation, undergoing dialysis or receiving chemotherapy; *N* = number of events in the 1–28 days following each dose in immunocompromised people; incidence rate ratios (IRR 99%CI) in the 1–28 days following vaccination compared to the baseline period are presented for pre-specified outcomes only where there were at least five events following a first, second or third dose in immunocompromised people. ITP, idiopathic or immune thrombocytopenic purpura; TIA, transient ischaemic attack; ICU, intensive care unit

Fifty-two of the 56 pre-specified adverse events had sufficient numbers of events to be included in the vaccine safety analysis. We found no significant increase in the incidence of any of the adverse events in the 1–28 days following a first, second or third dose of COVID-19 vaccine compared to the baseline period in immunocompromised people (Table 3, Fig. 4). In the analysis assessing the safety of each type of vaccine, we observed an increased risk of angioedema recorded in the 1–28 days following a first dose of ChAdOx1 compared to baseline in people who were immunocompromised (IRR 1.57 [99%CI 1.16–2.11]) (Additional file 6: Fig. S12). Following a third dose of BNT162b2, we observed an increased risk of multiple sclerosis (2.67 [1.03–6.95]), arrhythmia (1.29 [1.12–1.47]) and atrial fibrillation (1.29 [1.11–1.50]) compared to baseline. Additionally, we identified an increased risk of anaphylaxis following a third dose of mRNA-1273 (6.87 [1.64, 28.72]) (Additional file 6: Fig. S14). In the analysis comparing risks of each outcome in immunocompromised people with the general population, only the risk of multiple sclerosis was significantly higher in immunocompromised people (relative IRR 2.56 [99%CI 1.08–6.11]); however, the Bonferroni-corrected *p* value was not significant at the 1% level (corrected *p* = 0.22) (data not shown).

**Table 3:** Risk of unintended serious outcomes following COVID-19 vaccination in immunocompromised people*

	Dose 1		Dose 2		Dose 3	
Outcomes	***N﻿***	**IRR (99%CI)**	***N***	**IRR (99%CI)**	***N***	**IRR (99%CI)**
*Rheumatological*						
Vasculitis	217	0.95 (0.76, 1.20)	199	0.86 (0.69, 1.09)	101	0.75 (0.55, 1.02)
Systemic lupus erythematosus	10	0.52 (0.19, 1.42)	15	1.30 (0.58, 2.90)	10	0.78 (0.30, 1.99)
Rheumatoid arthritis	255	0.84 (0.68, 1.03)	290	0.91 (0.76, 1.10)	173	0.94 (0.74, 1.19)
Polymyalgia	159	0.85 (0.65, 1.12)	154	0.87 (0.67, 1.13)	88	0.78 (0.56, 1.10)
Inflammatory arthritis	152	0.91 (0.70, 1.19)	182	1.09 (0.86, 1.39)	75	0.92 (0.65, 1.31)
Scleroderma	5	0.44 (0.09, 2.06)	6	0.61 (0.16, 2.30)	< 5	–
Sjogren’s syndrome	10	0.94 (0.33, 2.73)	11	0.79 (0.30, 2.09)	< 5	–
Myositis	< 5	–	11	0.95 (0.38, 2.38)	9	1.41 (0.47, 4.18)
Ankylosing spondylitis	< 5	–	14	1.78 (0.67, 4.77)	5	2.21 (0.48, 10.11)
*Allergy*						
Anaphylaxis	32	1.06 (0.59, 1.89)	15	0.91 (0.43, 1.95)	12	1.19 (0.48, 2.98)
Angioedema	169	1.22 (0.96, 1.56)	135	0.98 (0.76, 1.27)	46	0.66 (0.43, 1.01)
Asthma	267	0.79 (0.66, 0.95)	265	0.88 (0.73, 1.05)	185	0.84 (0.68, 1.05)
*Liver disease*						
Acute liver injury	34	0.88 (0.49, 1.57)	18	0.40 (0.20, 0.82)	11	0.60 (0.25, 1.43)
Jaundice	38	0.64 (0.37, 1.10)	45	1.01 (0.62, 1.65)	26	1.19 (0.64, 2.22)
Autoimmune hepatitis	12	0.76 (0.29, 1.97)	9	0.77 (0.28, 2.11)	5	0.39 (0.09, 1.59)
Cholangitis	38	0.60 (0.35, 1.04)	40	0.64 (0.39, 1.04)	29	0.78 (0.44, 1.38)
Primary biliary cirrhosis	5	1.02 (0.21, 5.03)	< 5	–	< 5	–
*Blood disorders*						
Aplastic anaemia	37	0.76 (0.44, 1.30)	38	0.92 (0.55, 1.52)	15	0.81 (0.38, 1.72)
Haemolytic anaemia	9	1.51 (0.41, 5.52)	7	1.06 (0.30, 3.77)	< 5	–
ITP	97	1.17 (0.81, 1.68)	77	0.97 (0.67, 1.39)	53	1.24 (0.79, 1.95)
*Neuroinflammatory*						
Bell’s palsy	40	0.87 (0.53, 1.46)	30	0.79 (0.46, 1.37)	12	0.53 (0.23, 1.19)
Encephalitis	11	0.60 (0.24, 1.47)	11	0.58 (0.24, 1.40)	9	1.36 (0.48, 3.84)
Guillain–Barre syndrome	7	2.22 (0.46, 10.73)	< 5	–	< 5	–
Demyelinating disease	16	1.18 (0.52, 2.67)	10	0.85 (0.33, 2.18)	< 5	–
Multiple sclerosis	15	0.70 (0.31, 1.56)	14	0.70 (0.31, 1.55)	13	2.12 (0.81, 5.61)
Optic neuritis	10	1.33 (0.43, 4.06)	6	0.81 (0.22, 2.93)	< 5	–
*Cardiovascular*						
Myocarditis	7	0.43 (0.12, 1.54)	5	0.56 (0.14, 2.21)	< 5	–
Pericarditis	13	0.62 (0.26, 1.48)	18	0.95 (0.47, 1.95)	7	0.91 (0.29, 2.80)
Myocardial infarction	237	0.66 (0.53, 0.82)	234	0.68 (0.56, 0.84)	191	0.76 (0.61, 0.95)
Coronary heart disease	606	0.59 (0.51, 0.69)	555	0.64 (0.56, 0.73)	458	0.63 (0.55, 0.73)
Arrhythmia	778	0.74 (0.66, 0.84)	760	0.75 (0.67, 0.84)	568	0.79 (0.69, 0.90)
Atrial fibrillation	599	0.70 (0.60, 0.80)	563	0.69 (0.61, 0.79)	450	0.73 (0.63, 0.85)
Congestive cardiac failure	658	0.72 (0.63, 0.82)	699	0.73 (0.65, 0.82)	491	0.75 (0.65, 0.86)
Ischaemic stroke	323	0.68 (0.56, 0.82)	348	0.83 (0.70, 0.98)	214	0.74 (0.60, 0.91)
Haemorrhagic stroke	32	0.50 (0.25, 0.98)	27	0.52 (0.28, 0.98)	27	0.90 (0.50, 1.63)
Any stroke + TIA	356	0.71 (0.59, 0.84)	368	0.81 (0.69, 0.96)	244	0.77 (0.63, 0.94)
Subarachnoid haemorrhage	17	0.70 (0.31, 1.58)	24	0.74 (0.38, 1.44)	9	0.80 (0.29, 2.18)
Venous thromboembolism	434	0.74 (0.63, 0.87)	322	0.72 (0.60, 0.86)	196	0.70 (0.56, 0.87)
Arterial thrombosis	23	0.48 (0.24, 0.93)	28	0.64 (0.36, 1.15)	16	0.97 (0.45, 2.11)
*Inflammatory skin disease*						
Psoriasis	67	0.68 (0.47, 0.98)	79	1.04 (0.73, 1.47)	51	0.88 (0.57, 1.35)
Erythema nodosum	5	0.95 (0.24, 3.76)	< 5	–	< 5	–
Bullous eruption	16	0.90 (0.39, 2.08)	9	0.57 (0.21, 1.52)	6	0.70 (0.21, 2.34)
Bullous pemphigoid	23	1.07 (0.54, 2.16)	26	1.14 (0.61, 2.14)	12	0.85 (0.34, 2.08)
Pemphigus vulgaris	< 5	–	5	1.36 (0.29, 6.41)	< 5	–
*Autoimmune*						
Addison’s disease	53	0.66 (0.43, 1.00)	80	0.89 (0.62, 1.27)	39	0.83 (0.51, 1.34)
Pernicious anaemia	10	1.40 (0.47, 4.22)	14	1.23 (0.50, 3.02)	7	1.26 (0.38, 4.22)
Inflammatory bowel disease	97	0.82 (0.60, 1.13)	77	0.70 (0.50, 0.98)	35	0.58 (0.36, 0.95)
Thyroiditis	13	0.87 (0.37, 2.01)	9	0.61 (0.24, 1.59)	< 5	–
Coeliac disease	9	0.48 (0.17, 1.36)	13	1.18 (0.50, 2.79)	< 5	–
*Other outcomes*						
Acute renal failure	488	0.73 (0.63, 0.85)	417	0.67 (0.57, 0.77)	251	0.71 (0.58, 0.85)
Rhabdomyolysis	13	0.75 (0.30, 1.86)	11	0.68 (0.27, 1.68)	5	0.50 (0.14, 1.79)
Unplanned ICU admission	193	0.64 (0.51, 0.79)	275	0.68 (0.56, 0.82)	174	0.66 (0.53, 0.83)

* immunocompromised due to immunosuppressive drugs, organ transplantation, undergoing dialysis or receiving chemotherapy; *N* = number of events in the 1–28 days following each dose in immunocompromised people; incidence rate ratios (IRR 99%CI) in the 1–28 days following vaccination compared to the baseline period are presented for pre-specified outcomes only where there were at least five events following a first, second or third dose in immunocompromised people. *ITP* Idiopathic or immune thrombocytopenic purpura, *TIA* Transient ischaemic attack, *ICU* intensive care unit

## Discussion

This study provides a comprehensive analysis of real-world COVID-19 vaccine uptake, effectiveness and safety in clinically at-risk immunocompromised individuals due to immunosuppressive drug therapy or transplantation procedures using population-based data. We estimate that over 93% of immunocompromised people in England had received at least one COVID-19 vaccine dose by April 2022, but there were disparities in uptake across ethnic and socioeconomic groups. We also estimated COVID-19 vaccine effectiveness to be 91% against severe COVID-19 outcomes in immunocompromised people 2–6 weeks after the third dose. Lastly, we did not observe increased risks of 52 pre-specified safety outcomes following COVID-19 vaccination with any type of vaccine in immunocompromised people, but did find increased risks of five outcomes associated with specific types and doses of COVID-19 vaccine. However, COVID-19 vaccination did not result in a significantly higher risk of any other adverse events in immunocompromised people compared to the general population, suggesting there are generally no elevated safety concerns regarding their use in this clinically at-risk group.

We observed a decline in vaccine uptake with successive doses in immunocompromised people, which may present concern for rollout of future booster vaccines for clinically vulnerable populations. A contributory factor may be reduced visibility of the COVID-19 vaccination campaign within public perception in the context of the end of COVID-19 public health measures. We report significant and persistent health inequalities, both socioeconomic and ethnic, in COVID-19 vaccination uptake within the immunocompromised population in England, consistent with previous findings [8]. National data in England showed that ethnic disparities continued to persist in COVID-19 vaccine uptake during the 2023 booster rollouts [42]. These data suggest an urgent need for targeted communication to understand and address specific barriers to COVID-19 vaccine uptake within different ethnic and social groups of the wider immunocompromised population.

While immunogenicity studies have consistently demonstrated that COVID-19 vaccines result in a poorer immune response in immunocompromised people compared to people who are immunocompetent [43–45], COVID-19 vaccines still offer promising protection against severe outcomes. Evidence from other studies indicates vaccine effectiveness of 32 to 83% against COVID-19-related hospitalisation and 62 to 95% against COVID-19-related death in immunocompromised people [16–19, 27]. However, these studies noted some uncertainties in findings, primarily due to the limited number of cases observed in the immunocompromised people. Our findings align with similar studies using electronic health record data in the UK and USA reporting vaccine effectiveness against hospitalisation and/or death of around 90% following a third dose in people who are immunocompromised [27, 46]. Similarly, our study aligns with European research, demonstrating that heterologous booster vaccinations are 70–86% effective against severe illness, highlighting the benefits of booster doses in high-risk populations [47]. Consistent with the majority of studies, our results show that while COVID-19 vaccines provide a comparable level of protection, they are slightly less protective against hospitalisation and death in immunocompromised groups compared to the general population [46, 48].

The study population showed a higher proportion of COVID-19-related hospitalisations among immunocompromised individuals compared to the general population. Given that the outcome included outpatient hospital admissions and people with immunosuppressive conditions may have more frequent hospital visits and have a higher mean age, they may be more likely to have incidental COVID-19 hospital admissions [49]. Previous reports found that using broader definitions of COVID-19 hospitalisation resulted in lower vaccine effectiveness estimates due to outcome misclassification [50]. The estimates of vaccine effectiveness may have also been affected by residual confounding such as the timing of vaccinations corresponding to different circulating virus variants and shielding advice that was targeted towards clinically vulnerable groups in the UK [6]. We observed a progressive decrease in vaccine protection over time following each vaccine dose, supporting the need for booster doses in immunocompromised people. Further research including longer-term follow-up post-vaccination and assessing effectiveness of additional booster vaccine doses is required to determine the number of booster doses required and the optimum dosing interval.

Our findings showed that a first, second or third dose of any COVID-19 vaccine was not associated with occurrence of adverse events in the immunocompromised population, however, we when we stratified by vaccine type we identified increased risks of angioedema following a first dose with ChAdOx1, anaphylaxis following a third dose of mRNA-1273 and multiple sclerosis, arrhythmia and atrial fibrillation following a third dose of BNT162b2. The safety of COVID-19 vaccination in immunocompromised populations has been debated due to previous studies linking COVID-19 vaccines to some immune-related adverse events, such as Guillain–Barre syndrome, myocarditis and immune thrombocytopenia, but these have generally been very rare occurrences, which although demonstrated to have statistically significant link to vaccination, have higher risk of occurrence following COVID-19 infection [22–24, 51]. A recent comprehensive study further supports the safety profile of COVID-19 vaccines, finding background rates for 41 adverse events of special interest in the European population to be low, although incidence rates were higher in people with underlying conditions, including immunocompromised groups, across all outcomes [52]. Allergic reactions are monitored following all vaccinations but are usually mild and reported at low rates [53]. While increased risks of anaphylaxis and angioedema were identified following COVID-19 vaccination with ChAdOx1 and mRNA-1273 in immunocompromised people, these risks were not significantly higher than observed in the general population. However, special considerations to prevent and manage allergic reactions following vaccination should continue to be taken in immunocompromised people to ensure their safety [53]. A recent meta-analysis and systematic review reported a small increased risk of cardiac arrhythmias following mRNA COVID-19 vaccines, as reported in this study with increased risks of arrhythmia and atrial fibrillation observed following a third dose of BNT172b2 in both immunocompromised people and the general population [54]. There have also been case reports of multiple sclerosis presentation [55–57] following COVID-19 vaccine exposure; however, there is little evidence for vaccination causing new-onset disease. Furthermore, multiple sclerosis is a complex condition typically diagnosed over a period of time, involving referral to a neurological specialist [58]; therefore, any diagnoses recorded within 28 days following vaccination are unlikely to be directly associated with the vaccine.

### Strengths and limitations

To our knowledge, this is the largest study of COVID-19 vaccine uptake, safety and effectiveness in immunocompromised populations. There are some limitations in our study. Firstly, we categorised our target population using medical records with diagnostic codes or drug prescriptions related to having an immunocompromised condition for a 6-month period before the study start date (24 months for bone marrow transplant patients), which may have led to misclassification biases, and the population does not include all immunocompromised conditions, such as people with HIV conditions alone. Furthermore, immune-modifying drugs were broadly categorised, including the use of steroids. We recognise the potential variability in immunosuppression levels, especially among individuals with only occasional or acute use of oral steroids, which might lead to over-estimation of vaccine effectiveness, as not all those in the immunosuppressed group may have severe immunosuppression. This may also have uncertain impact on safety estimates. Second, due to a limited number of severe outcome cases after the fourth dose within the immunocompromised population, our assessment of vaccine effectiveness and safety was primarily centred on the effectiveness and safety of the vaccine for the initial three doses, as reflected in our subgroup analyses. Third, despite conforming with pre-defined definitions used in national reports [36], classification of outcomes according to presence of a positive COVID-19 test within a specified time period before a hospital admission (within 14 days) or during a hospital admission or death (within 28 days) may misclassify cases where COVID-19 is an incidental finding rather than the primary reason for admission or death. This approach faces the limitation of not being able to distinctly separate hospital-acquired infections, making it hard to determine if COVID-19 was the main cause of admission or death. Fourth, we employed the self-controlled cases series method to compare vaccine safety between immunocompromised individuals and the general population, controlling for internal factors by using subjects as their own controls. However, the method has limitation in adjusting for external confounders; furthermore, we are unable to exclude exacerbations of pre-existing conditions from adverse events which necessitates cautious interpretation, and longer time windows of evaluation may be more suited for some types of adverse events. Fifth, we did not have data available on number of SARS-CoV-2 tests taken by each person or the underlying SAR-CoV-2 variant for each positive SARS-CoV-2 test, so we were unable to adjust for these factors in the analysis.

## Conclusions

We found high COVID-19 vaccine uptake in immunocompromised people in England albeit with disparities across different ethnic and socioeconomic groups. Our findings show that two to three doses of any COVID-19 vaccine effectively protect against COVID-19-related hospitalisation, ICU admission and death in immunocompromised people, with effectiveness and safety profiles comparable to that of the general population.

While acknowledging study limitations and confounding factors, our findings underscore the importance of ongoing vaccination prioritisation for immunocompromised individuals, who face a greater risk from COVID-19 compared to the general population, to maximise protection against severe outcomes. This prioritisation is essential to ensure that vaccination efforts remain responsive to emerging challenges and continues to protect those at highest risk.

### Supplementary Information

Additional file 1: Definition and code groups of immunosuppressed patients.

Additional file 2: List of adverse events of special interest for vaccine safety.

Additional file 3: Vaccine uptake in immunocompromised patients: statistical analysis plan.

Additional file 4: Vaccine effectiveness in immunocompromised patients: statistical analysis plan.

Additional file 5: Vaccine safety in immunocompromised patients: statistical analysis plan.

Additional file 6: Figure S1: Kaplan–Meier curves of first to fourth COVID-19 vaccine uptake in the immunocompromised and the general population; Figure S2: Kaplan–Meier curves of first to fourth COVID-19 vaccine uptake in the immunocompromised and general population among people aged 60 and above; Figure S3: COVID-19 vaccine uptake by dose in the general and immunocompromised population; Figure S4: COVID-19 vaccine uptake by dose and vaccine types in the immunocompromised population; Figure S5: COVID-19 vaccine uptake by dose and subgroups in the immunocompromised population; Figure S6: COVID-19 vaccine uptake by dose and ethnicity in the immunocompromised population; Figure S7: COVID-19 vaccine uptake by dose and deprivation quintile in the immunocompromised population; Figure S8: Adjusted vaccine effectiveness against COVID-19-related hospitalisation by vaccine types in the immunocompromised population; Figure S9: Adjusted vaccine effectiveness against COVID-19-related hospitalisation in the immunocompromised population by periods of different dominant variant in the UK; Figure S10: Adjusted vaccine effectiveness against COVID-19-related death in the immunocompromised population by periods of different dominant variant in the UK; Figure S11: Risk of serious outcomes following a first, second or third dose of COVID-19 vaccine in immunocompromised people relative to people who are not immunocompromised; Figure S12: Risk of serious outcomes following a first dose of ChAdOx1 or BNT162b2 vaccine in immunocompromised people; Figure S13: Risk of serious outcomes following a second dose of ChAdOx1 or BNT162b2 vaccine in immunocompromised people; Figure S14: Risk of serious outcomes following a third dose of BNT162b2 or mRNA-1273 vaccine in immunocompromised people; Table S1: Multivariable Cox regression analyses of COVID-19 vaccine uptake in the immunocompromised population; Table S2: Adjusted vaccine effectiveness against COVID-19 outcomes in the immunocompromised population by vaccine types and periods of different dominant variant in the UK; Table S3: Demographic characteristics of the immunocompromised population according COVID-19 outcomes (*n* = 583,541).

## Data Availability

To guarantee the confidentiality of personal and health information, only the authors have had access to the data during the study in accordance with the relevant licence agreements. Access to the QResearch data is according to the information on the QResearch website (www.qresearch.org).

## Abbreviations

95%CI: 95% Confidence interval
99%CI: 99% Confidence interval
BMI: Body mass index
BNF: British National Formulary
COVID-19: Coronavirus disease
HES: Hospital Episode Statistics
HIV: Human immunodeficiency virus
HR: Hazard ratio
ICD10: International Classification of Diseases 10th Revision
ICU: Intensive care unit
IRR: Incidence rate ratio
JCVI: Joint Committee on Vaccination and Immunisation
MICE: Multiple imputation using chained equations
NCRAS: National Cancer Registration and Analysis Service data
NHS England: National Health Service England
NIMS: National Immunisation Management System
ONS: Office for National Statistics
OR: Odds ratio
SARS-CoV-2: Severe acute respiratory syndrome coronavirus 2
SD: Standard deviation
SGSS: Second Generation Surveillance System
