# Supplementary material for: Uptake, effectiveness and safety of COVID-19 vaccines in individuals at clinical risk due to immunosuppressive drug therapy or transplantation procedures: a population-based cohort study in England
Source: BMC Med. 2024 Jun 10;22:237. doi: 10.1186/s12916-024-03457-1 (PMC11165729; doi:10.1186/s12916-024-03457-1)
Supplement: Supplementary file 1 — Additional file 1: Definition and code groups of immunosuppressed patients. [file 12916_2024_3457_MOESM1_ESM.docx]

**Supplementary Information: Uptake, effectiveness and safety of COVID-19 vaccines in the immunocompromised population: A population-based cohort study in England**

Additional file 1: Definition and code groups of immunosuppressed patients.

**Transplant/procedures (before 1^st^ December 2020)**

1. Liver transplant
2. Kidney transplant
3. Solid organ transplant
4. Bone marrow transplant (within 24 months)
5. Renal dialysis

**Drugs – taken anytime from 1^st^ June 2020 to 1^st^ December 2020**

1. Dexamethasone
2. Drugs that affect the immune response
3. Immune-modifying monoclonal antibodies
4. Immunosuppressants for transplants
5. Oral Steroids
6. Other Immune Modulators
7. Chemotherapy

|  | **Code group ID** ([QCode Group Library - QResearch](https://www.qresearch.org/data/qcode-group-library/)) | | | |
| --- | --- | --- | --- | --- |
|  | **Read/SNOMED** | **ICD-10** | **OPCS (procedures)** | **Drug group** |
| **Transplants** |  |  |  |  |
| Liver | 7551 |  |  |  |
| Renal |  | 2772 | 2774 |  |
| Solid organ |  |  | 7571 |  |
| Bone marrow | 7795 |  | 7568 |  |
|  |  |  |  |  |
| **Dialysis and renal replacement therapy** |  |  |  |  |
| Dialysis |  |  | 2776 |  |
| Renal replacement therapy (dialysis or transplant) | 2223 | 2784 | 2777 |  |
|  |  |  |  |  |
| **Chemo and radiotherapy** |  |  |  |  |
| Chemotherapy |  |  | 2878 | 15207 /  15208 /  15209 |
| Radiotherapy |  |  | 2916 |  |
| Chemo/radiotherapy |  |  | 7739 |  |
|  |  |  |  |  |
| **Drugs** |  |  |  |  |
| Dexamethasone |  |  |  | 13839 |
| Oral steroids |  |  |  | 6454 |
| Immune-modifying monoclonal antibodies (mAbs) |  |  |  | 6467 |
| Immunosuppressants for transplants |  |  |  | 7572 |
| Other immune-modifying drugs |  |  |  | 6455/ 6386 |

**Diagram of the subgroups included in the analysis (n=** **583,541)**


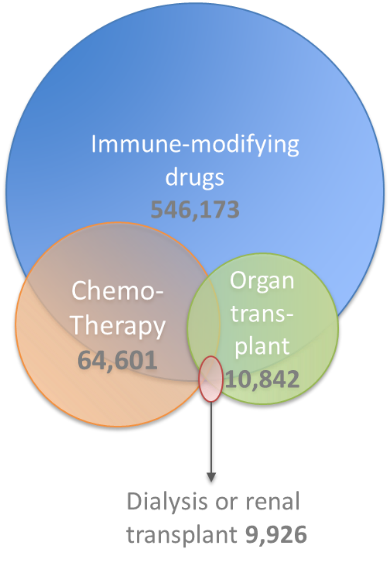


- Immune-modifying drugs
- Chemotherapy
- Organ transplant procedures
- Dialysis or renal transplant
