# Supplementary material for: Uptake, effectiveness and safety of COVID-19 vaccines in individuals at clinical risk due to immunosuppressive drug therapy or transplantation procedures: a population-based cohort study in England
Source: BMC Med. 2024 Jun 10;22:237. doi: 10.1186/s12916-024-03457-1 (PMC11165729; doi:10.1186/s12916-024-03457-1)
Supplement: Supplementary file 2 — Additional file 2: List of adverse events of special interest for vaccine safety. [file 12916_2024_3457_MOESM2_ESM.docx]

**Supplementary Information: Uptake, effectiveness and safety of COVID-19 vaccines in the immunocompromised population: A population-based cohort study in England**

Additional file 2: List of adverse events of special interest for vaccine safety

| **Type of outcome** | **Individual outcomes** |
| --- | --- |
| Rheumatological conditions | - Rheumatoid arthritis - Systemic lupus erythematosus - Vasculitis - Ankylosing spondylitis - Inflammatory arthritis - Scleroderma - Sjogren's syndrome - Goodpasture syndrome - Myositis - Polymyalgia |
| Liver disease | - Acute liver injury - Jaundice - Autoimmune hepatitis - Cholangitis - Primary biliary cirrhosis |
| Blood disorders | - Aplastic anaemia - Idiopathic thrombocytopenic purpura - Immune haemolytic anaemia |
| Neuroinflammatory disorders | - Bell's Palsy - Encephalitis - Meningitis - Myelitis - Guillain-Barré syndrome - Demyelinating disorders - Multiple sclerosis - Optic neuritis |
| Cardiovascular disease | - Myocarditis - Pericarditis - Myocardial infarction - Coronary heart disease - Arrhythmia - Atrial fibrillation or flutter - Congestive cardiac failure - Ischaemic stroke - Haemorrhagic stroke - Transient ischaemic attack - Subarachnoid haemorrhage - Venous thromboembolism - Arterial thrombosis |
| Inflammatory skin conditions | - Pemphigus vulgaris - Bullous pemphigoid/eruption - Erythema nodosum - Psoriasis |
| Other autoimmune disorders | - Addison's adrenal insufficiency - Pernicious anaemia - Multisystem inflammatory syndrome - Inflammatory bowel disease - Acute and chronic thyroiditis - Coeliac disease |
| Allergy-related conditions | - Anaphylaxis - Angioedema - Asthma |
| Other outcomes that do not fit into above categories | - Acute renal failure - Rhabdomyolysis - Unplanned ICU admission - Sudden death |
