# Supplementary material for: Uptake, effectiveness and safety of COVID-19 vaccines in individuals at clinical risk due to immunosuppressive drug therapy or transplantation procedures: a population-based cohort study in England
Source: BMC Med. 2024 Jun 10;22:237. doi: 10.1186/s12916-024-03457-1 (PMC11165729; doi:10.1186/s12916-024-03457-1)
Supplement: Supplementary file 3 — Additional file 3: Vaccine uptake in immunocompromised patients: statistical analysis plan. [file 12916_2024_3457_MOESM3_ESM.docx]

**Supplementary Information: Uptake, effectiveness and safety of COVID-19 vaccines in the immunocompromised population: A population-based cohort study in England**

Additional file 3: Vaccine uptake in immunocompromised patients: Statistical analysis plan

**Objectives**

***Primary Objective***

Determine factors associated with the uptake of COVID-19 vaccination in people with immunocompromised conditions

***Secondary Objectives***

Determine factors associated with the uptake of COVID-19 vaccination in:

- Immunocompromised populations
- The general population

**Study Design**

Cohort study

*Inclusion criteria*

All patients aged over 12 years with data on COVID-19 vaccination

The study period will be 1^st^ December 2020 to the latest available data download.

Dates for each of the COVID-19 vaccine doses will start from

- Vaccine dose 1 – 1 Dec 2020
- Vaccine dose 2 – 1 March 2021
- Vaccine dose 3 (booster) - 14^th^ Sept 2021
- Vaccine dose 4 – spring booster for most vulnerable – 1^st^ Jan 2022

*Data source*

Our main analyses will be based on the QResearch database linked to the following datasets to improve ascertainment of exposures, confounders and outcomes:

- Pillar 1 and 2 testing data (PHE SGSS)
- Civil registration data (NHS Digital)
- HES care data (NHS Digital)
- Intensive Care National Audit and Research Centre Case Mix Programme (ICNARC)
- Cancer registry, SACT & Radiotherapy (PHE)
- COVID-19 Vaccine uptake data from the National Immunisation Database (NIMS, NHS Digital)
- COVID-19 vaccination adverse events (NIMS, NHS Digital)

*Outcomes*

Primary Outcome: Uptake of COVID-19 vaccine (one or more doses) in people with immunocompromised conditions.

Secondary Outcomes:

- Uptake of 3^rd^ vaccine dose in people with immunocompromised conditions
- Uptake of 4^th^ vaccine dose in people with immunocompromised conditions

Predictors of one of more doses of vaccine in people with immunocompromised conditions

**Statistical analyses**

*Descriptive statistics*

Tables showing uptake of each dose of vaccine (0-4 doses) by demographics (age, sex, categories of BMI (underweight, normal weight as reference, overweight, obese), ethnicity (white as reference group) and socioeconomic status, (Q1 as reference), geographic region of the UK (London as reference) and type of immunocompromised conditions (receiving immune-modifying drugs as reference).

Bar charts showing uptake of COVID-19 vaccine doses 1-4 by age, ethnicity, socioeconomic status (IMD quintile) and geographic region of the UK.

Kaplan Meyer curves showing time to vaccine uptake by age, ethnicity, socioeconomic status (IMD quintile) and geographic region of the UK.

*Covariates*

- Townsend quintile of deprivation
  - Q1 (Most affluent) to Q5(most deprived)
- Ethnicity
  - White, Indian, Pakistani, Chinese, Bangladeshi, Other Asian, Black Caribbean, Black African, Other, Not recorded
- Regions
  - East Midlands, East of England, North East, North West, South Central, South East, South West, West Midlands, Yorkshire & Humber
- Immunocompromised conditions
  - Organ transplant procedures (solid organ, liver or bone marrow),
  - Renal transplant or dialysis
  - Receiving immune-modifying drugs
  - Receiving chemotherapy

*Identifying predictors of vaccine uptake in people with blood cancer* *and immunocompromised populations*

Analyses will be restricted to the populations of interest (those with immunocompromised conditions). Cox regression will be used to identify factors associated with vaccine uptake (or non-uptake).

Cox regression to explore time to vaccine uptake reporting adjusted hazard ratios for each vaccine dose. Dates for vaccine uptake will be number of days from when the specific vaccine dose first became available. People will be censored from the analysis when they leave the practice, dies or on the latest date when data are available. We will check for proportional hazards and extend to using Royston-Palmar models to account for time varying hazard ratios if proportional hazards assumption not valid.

The primary analysis will be repeated using the following methods to check that results are consistent with the primary analysis (Cox regression):

*Covariates*

Model: Adjusted for age, sex, BMI, ethnicity, socioeconomic status (IMD quintile), geographic region of the UK and QCOVID comorbidities (ref 32 in main text).

Where there are sufficient data, vaccine uptake in each type of immunocompromised conditions (organ transplant procedures [solid organ, liver or bone marrow], renal transplant or dialysis, receiving immune-modifying drugs or receiving chemotherapy) will be carried out. Analyses by individual immunocompromised conditions type will only be conducted where there are more than 2500 individuals to ensure that there are a minimum of 10 subjects per parameter (ref 33 in main text).

*Sensitivity analyses*

Multilevel logistic regression models will be used to examine the effect of GP practice & region on uptake.

*Subgroup analyses*

We will explore the association between deprivation and ethnicity in vaccine uptake by conducting analyses within each ethnicity and within each quintile of deprivation using cox regression models.
