# Supplementary material for: Uptake, effectiveness and safety of COVID-19 vaccines in individuals at clinical risk due to immunosuppressive drug therapy or transplantation procedures: a population-based cohort study in England
Source: BMC Med. 2024 Jun 10;22:237. doi: 10.1186/s12916-024-03457-1 (PMC11165729; doi:10.1186/s12916-024-03457-1)
Supplement: Supplementary file 4 — Additional file 4: Vaccine effectiveness in immunocompromised patients: statistical analysis plan. [file 12916_2024_3457_MOESM4_ESM.docx]

**Supplementary Information: Uptake, effectiveness and safety of COVID-19 vaccines in the immunocompromised population: A population-based cohort study in England**

Additional file 4: Vaccine effectiveness in immunocompromised patients: Statistical analysis plan

**Aim**

To determine the effectiveness of COVID-19 vaccines in people with immunocompromised conditions.

**Objectives**

- Examine the odds of COVID-19-related outcomes after vaccination with one, two, or three vaccines in people with immunocompromised conditions (within immunocompromised group comparison)
- Examine the odds of COVID-19-related outcomes after vaccination with one, two, or three vaccines in people with blood cancer compared with the general population (between immunocompromised group comparison)

**Primary analysis**

Nested matched case-control design including both vaccinated and unvaccinated populations

**Outcomes**

The outcomes of interest are the occurrence and odds of the below COVID-19-related severe events following one, two, or three doses of vaccines compared to those who have not received the vaccine.

- COVID-19 Hospitalisation (hospitalisation within 14 days of testing COVID-19 positive)
- COVID-19-related ICU admission (admission to intensive care during a COVID-19 hospitalisation)
- COVID-19-related Death (death within 28 days of COVID-19 positive or COVID-19 on death certificate)

Cases will be patients who have had COVID-19 hospitalisation or COVID-19-related death, and will be matched to controls who did not experience the outcome of interest. Patients who experienced COVID-19-related ICU admission will be a subset of the COVID-19 hospitalisation cases and therefore will not be matched separately.

**Exposure definition**

*COVID-19 vaccination:*

It is expected to take 14 days for an immune response to the vaccine to develop (ref 34 in main text). We will therefore consider the population exposed 14 days after vaccination. For the analysis we will consider the exposure periods as used in previous research, where there are sufficient numbers (ref 32 & 35 in main text). For the analysis, we will define *vaccination status* considering one to four doses of any COVID-19 vaccine and the indicative time periods since vaccination as follows, although these may be amended after assessing the distribution of the data:

- Unvaccinated
- Days (weeks) after 1^st^ dose
- 0-13 days (<2 weeks)
- 14-27 days (2 - <4 weeks)
- 28-41 days (4 - <6 weeks)
- 42+ days (≥6 weeks)
- Days (weeks) after 2^nd^ dose
- 0-13 days (<2 weeks)
- 14-41 days (2 - <6 weeks)
- 42-97 days (6 - <14 weeks)
- 98-153 days (14 - <22 weeks)
- 154-181 days (22 - <26 weeks)
- 182-272 days (26 - <39 weeks)
- 273+ days (≥39 weeks)
- Days (weeks) after 3^rd^ dose
- 0-13 days (<2 weeks)
- 14-41 days (2 - <6 weeks)
- 42-97 days (6 - <14 weeks)
- 98-125 days (14 - <18 weeks)
- 126-153 days (18 - <22 weeks)
- 154-181 days (22 - <26 weeks)
- 182-272 days (26 - <39 weeks)
- 273+ days (≥39 weeks)

The following vaccine types will also be considered, where numbers are sufficient:

- BNT162b2
- ChAdOx1
- mRNA-1273

**Matching**

All cases who had the outcome will be matched with 10 controls who did not have the outcome on that date by matching on age, sex, calendar date and GP practice using incidence density sampling (ref 35 in main text). We will remove GP practice from the matching criteria if there is not a sufficient number of controls to match to each case in the blood cancer group.

We will undertake matching in the cohort of immunocompromised patients and the general population separately.

**Statistical analysis**

People will enter the cohort on 01/12/2020 (start date) and censored on the earliest date of outcome of interest (hospitalisation, ICU admission, or death) or end of the study period 11/04/2022 (end date). Individuals with the outcome of interest before the study start date will be excluded from the primary analysis.

Conditional logistic regression models will be used to report odds ratios and 95% confidence intervals between vaccinated and unvaccinated groups, adjusting for covariates as listed below. Subgroups of immunocompromising conditions will also be examined using the same methods described above, provided there is sufficient data for analysis.

We will conduct stratified analyses by calendar time to account for different SARS-CoV-2 variants circulating in the UK. We will split calendar time into the following intervals (ref 36 in main text):

- Alpha: 18 Dec 2020 – 17 May 2021
- Delta: 18 May 2021 – 19 Dec 2021
- Omicron BA.1: 20 Dec 2021 – 1 March 2022

*Covariates*

- Previous SARS-CoV-2 infection (ref 37 in main text).
- Socioeconomic status as measured by the Townsend score
- Ethnicity
- BMI (consider multiple imputation for missing values)
- Co-morbidities: 36 underlying clinical conditions identified using the QCOVID risk algorithm, where available in the dataset (ref 32 in main text). Co-morbidities will be grouped as below and included in the model by the following categories:
- Cardiovascular conditions (atrial fibrillation, heart failure, stroke, peripheral vascular disease, coronary heart disease, congenital heart disease)
- Diabetes (type 1 and type 2)
- Respiratory conditions (asthma, rare respiratory conditions (cystic fibrosis, bronchiectasis, or alveolitis), chronic obstructive pulmonary disease, pulmonary hypertension or pulmonary fibrosis)
- Cancer (lung or oral cancer, marrow transplant, radiotherapy)
- Neurological conditions (cerebral palsy, Parkinson’s disease, rare neurological conditions (motor neurone disease, multiple sclerosis, myasthenia, Huntington’s chorea), epilepsy, dementia, learning disability, severe mental illness)
- Other conditions (liver cirrhosis, osteoporotic fracture, rheumatoid arthritis or systemic lupus erythematosus, sickle cell disease, venous thromboembolism, solid organ transplant, renal failure (CKD3, CKD4, CKD5, with or without dialysis or transplant))
- Medications (prescriptions from general practitioner in previous six months for oral steroids, long acting β agonists or leukotrienes, immunosuppressants)
- Chemotherapy – (compare active chemotherapy during study period, previous chemotherapy in 6 months prior to study start date, and those not treated with chemotherapy from 6 months before the study start date)
