# Supplementary material for: Uptake, effectiveness and safety of COVID-19 vaccines in individuals at clinical risk due to immunosuppressive drug therapy or transplantation procedures: a population-based cohort study in England
Source: BMC Med. 2024 Jun 10;22:237. doi: 10.1186/s12916-024-03457-1 (PMC11165729; doi:10.1186/s12916-024-03457-1)
Supplement: Supplementary file 5 — Additional file 5: Vaccine safety in immunocompromised patients: statistical analysis plan. [file 12916_2024_3457_MOESM5_ESM.docx]

**Supplementary Information: Uptake, effectiveness and safety of COVID-19 vaccines in the immunocompromised population: A population-based cohort study in England**

Additional file 5: Vaccine safety in immunocompromised patients: Statistical analysis plan

**Study Design**

We will utilise self-controlled case-series design.

**Statistical analysis**

We will explore the incidence rate of potential post-vaccine adverse events in several groups, including people with immunocompromised conditions.

The outcomes to be assessed will be post-vaccination occurrence of any of adverse events of special interest, listed in Table 1. These include events previously highlighted in relation to vaccine safety or because they have been identified as specific events in the Oxford Vaccine Protocol or are events which need to be monitored by the European Medicines Agency (EMA) Designated Medical Event, FDA, the UK’s Medicines Health Regulatory Authority vaccine clinical trials, post-marketing surveillance and the emerging scientific literature.

Cases are defined as individuals aged 12 years and above that have experienced an adverse event of interest after the 1st December 2020.

Exposures of interest are a first, second or third dose of each COVID-19 vaccine and a SARS-CoV-2 infection defined as SARS-CoV-2 positive test or recorded COVID-19 diagnosis.

A self-controlled case series approach, previously established to assess adverse events to vaccination (ref 38 & 39 in main text), will be used to estimate whether the risks of complications within 28 days following a COVID-19 vaccine or a SARS-CoV-2 positive test are different in people with immunocompromised conditions compared with people without these conditions. In this approach, we will determine the relative incidence of the outcome of interest for exposed time periods (i.e. following vaccination) compared to unexposed periods in individuals who have the outcome of interest in two subgroups: those with a immunocompromised conditions on 1st December 2020 and those without. Inference is within individuals and hence covariates which do not change over the study period are implicitly controlled for.

Observation time for each case will be divided into ‘baseline’, ‘pre-vaccine’ and ‘post-vaccine’ periods. These periods will be: -29-1 days prior to vaccination, the day of vaccination and 1-7; 8-14; 15-21; 22-28 days post vaccination days, with remainder of observation time contributing to the ‘baseline’. The post-exposure timeframe broken into 7 day blocks to account for time-varying effects, i.e. potentially non-uniform risks of complications after vaccine administration.

Fixed-effects Poisson regression models will be used to estimate the incident rate ratios (IRR, with 95% confidence intervals) for each time block. Immunocompromised status will be included as interaction terms to explore whether IRRs are significantly different in these groups compared to the wider population. For example, the presence of a recorded of immunocompromised conditions will be a binary variable (0/1), which will be interacted with the exposure variable (vaccination) in the Poisson model. Models will also include terms for positive SARS-CoV-2 tests, as COVID-19 may cause some of the complications of interest and a two-week period to adjust for seasonal effect and temporal changes in the outcome incidence due to the pandemic. Separate models will be developed for each specific adverse event.

**Sensitivity analyses**

1. Restricting the models to those without a record of a positive SARS-CoV-2 test during the study period
2. Excluding those who died during the study period
3. Excluding those with the outcome of interest recorded in the month prior (1^st^ – 30^th^ November 2020)
4. Excluding those with the outcome of interest recorded in the two years prior (1^st^ December 2018 – 30^th^ November 2020) to the study start date
5. No censoring for deaths due to outcome
6. Defining start date as date of 1^st^ dose, with no censoring for deaths due to outcome
7. Defining start date as date of 2^nd^ dose, with no censoring for deaths due to outcome
8. Defining start date as date of 3^rd^ dose, with no censoring for deaths due to outcome

**Table 1 - Vaccine adverse events**

| **Category** | **Event** | **QResearch Read/SNOMED Code group ID** | **QResearch**  **ICD-10 Code Group ID** |
| --- | --- | --- | --- |
| General | Sudden death | n/a | n/a |
|  | Unplanned ICU admission | n/a | n/a |
|  | Adult Respiratory Distress Syndrome | n/a | n/a |
| Autoimmune | Anaphylaxis | 301 | 13058 |
|  | Angioedema | 13079 | 13080 |
|  | Cholangitis | 6082 | 13495 |
|  | Rhabdomyolysis | 606 | 2740 |
|  | Addison’s disease | 6088 | 13484 |
|  | Inflammatory bowel disease |  |  |
|  | Pernicious anaemia | 75 | 13485 |
|  | Vasculitis | 7582 | 13498 |
|  | Scleroderma | 71 | 13492 |
|  | Sjogren’s syndrome | 72 | 13487 |
|  | Thyroiditis | 6148 | 11312 |
|  | Coeliac disease | 47 | 1979 |
| Musculoskeletal | Spondyloarthritis and ankylosing spondylitis | 6090 | 13493 |
|  | Systemic lupus erythematosus | 70 | 1976 |
|  | Rheumatoid arthritis | 68 | 1975 |
|  | Polymyalgia | 7911 | 13491 |
|  | Inflammatory arthritis | 16903 | 16894 |
|  | Myositis | 73 | 13490 |
| Gastrointestinal | Acute liver injury or hepatic failure | 1333 | 13061 |
|  | Jaundice | 1841 | 13060 |
|  | Autoimmune hepatitis | 6149 | 13065 |
|  | Primary biliary cirrhosis | 6089 | 13483 |
| Renal | Acute kidney injury | 2742 | 2466 |
| Blood | Aplastic anaemia/pancytopenia | 1313 | 13073 |
|  | Haemolytic anaemia | 1314 | 13062 |
|  | ITP | 6154 | 13074 |
| Neurological | Bell’s palsy | 1316 | 13069 |
|  | Encephalitis and myelitis | 13078 | 13071 |
|  | Guillain Barre syndrome | 1318 | 13066 |
|  | Demyelinating disease | 14239 | 14240 |
|  | Multiple sclerosis | 38 | 11193 |
|  | Optic neuritis | 13082 | 13083 |
| Skin | Bullous eruption including Stevens Johnson | 619 | 3305 |
|  | Autoimmune bullous skin diseases, including pemphigus, pemphigoid and dermatitis herpetiformis | 2423 or 6139 |  |
|  | Erythema nodosum | 13499 | 13500 |
|  | Psoriasis | 1896 | n/a |
| Cardiovascular | Myocardial infarction | 20 | 1950 |
|  | Arrythmias | 1205 | 24444 |
|  | Myocarditis or pericarditis | 2220 | 13084 |
|  | Coronary heart disease | 19 | 1928 |
|  | Atrial fibrillation | 24 | 11204 |
|  | Congestive cardiac failure | n/a | 1936 |
|  | Ischaemic stroke | 272 | 1929 |
|  | Haemorrhagic stroke | n/a | 14698 |
|  | Stroke | 272 | 1929 |
|  | Subarachnoid haemorrhage | 14242 | 14243 |
|  | Venous thromboembolism | 368 | 1935 |
|  | Arterial thrombosis | 13917 | 13916 |
