# Supplementary material for: Uptake, effectiveness and safety of COVID-19 vaccines in individuals at clinical risk due to immunosuppressive drug therapy or transplantation procedures: a population-based cohort study in England
Source: BMC Med. 2024 Jun 10;22:237. doi: 10.1186/s12916-024-03457-1 (PMC11165729; doi:10.1186/s12916-024-03457-1)
Supplement: Supplementary file 6 — Additional file 6: Figure S1: Kaplan–Meier curves of first to fourth COVID-19 vaccine uptake in the immunocompromised and the general population; Figure S2: Kaplan–Meier curves of first to fourth COVID-19 vaccine uptake in the immunocompromised and general population among people aged 60 and above; Figure S3: COVID-19 vaccine uptake by dose in the general and immunocompromised population; Figure S4: COVID-19 vaccine uptake by dose and vaccine types in the immunocompromised population; Figure S5: COVID-19 vaccine uptake by dose and subgroups in the immunocompromised population; Figure S6: COVID-19 vaccine uptake by dose and ethnicity in the immunocompromised population; Figure S7: COVID-19 vaccine uptake by dose and deprivation quintile in the immunocompromised population; Figure S8: Adjusted vaccine effectiveness against COVID-19-related hospitalisation by vaccine types in the immunocompromised population; Figure S9: Adjusted vaccine effectiveness against COVID-19-related hospitalisation in the immunocompromised population by periods of different dominant variant in the UK; Figure S10: Adjusted vaccine effectiveness against COVID-19-related death in the immunocompromised population by periods of different dominant variant in the UK; Figure S11: Risk of serious outcomes following a first, second or third dose of COVID-19 vaccine in immunocompromised people relative to people who are not immunocompromised; Figure S12: Risk of serious outcomes following a first dose of ChAdOx1 or BNT162b2 vaccine in immunocompromised people; Figure S13: Risk of serious outcomes following a second dose of ChAdOx1 or BNT162b2 vaccine in immunocompromised people; Figure S14: Risk of serious outcomes following a third dose of BNT162b2 or mRNA-1273 vaccine in immunocompromised people; Table S1: Multivariable Cox regression analyses of COVID-19 vaccine uptake in the immunocompromised population; Table S2: Adjusted vaccine effectiveness against COVID-19 outcomes in the immunocompromised popul [file 12916_2024_3457_MOESM6_ESM.docx]

**Supplementary Information: Uptake, effectiveness and safety of COVID-19 vaccines in the immunocompromised population: A population-based cohort study in England**

Additional file 6:

Figure S1 – Kaplan-Meier curves of first to fourth COVID-19 vaccine uptake in the immunocompromised and the general population

Figure S2 – Kaplan-Meier curves of first to fourth COVID-19 vaccine uptake in the immunocompromised and general population among people aged 60 and above.

Figure S3 – COVID-19 vaccine uptake by dose in the general and immunocompromised population

Figure S4 – COVID-19 vaccine uptake by dose and vaccine types in the immunocompromised population

Figure S5 – COVID-19 vaccine uptake by dose and subgroups in the immunocompromised population

Figure S6 – COVID-19 vaccine uptake by dose and ethnicity in the immunocompromised population

Figure S7 – COVID-19 vaccine uptake by dose and deprivation quintile in the immunocompromised population

Figure S8 – Adjusted vaccine effectiveness against COVID-19-realted hospitalisation by vaccine types in the immunocompromised population

Figure S9 – Adjusted vaccine effectiveness against COVID-19-realted hospitalisation in the immunocompromised population by periods of different dominant variant in the UK

Figure S10 – Adjusted vaccine effectiveness against COVID-19-realted death in the immunocompromised population by periods of different dominant variant in the UK

Figure S11 – Risk of serious outcomes following a first, second or third dose of COVID-19 vaccine in immunocompromised people relative to people who are not immunocompromised

Figure S12 – Risk of serious outcomes following a first dose of ChAdOX1 or BNT162b2 vaccine in immunocompromised people

Figure S13 – Risk of serious outcomes following a second dose of ChAdOX1 or BNT162b2 vaccine in immunocompromised people

Figure S14 – Risk of serious outcomes following a third dose of BNT162b2 or mRNA-1273 vaccine in immunocompromised people

Table S1 – Multivariable Cox regression analyses of COVID-19 vaccine uptake in the immunocompromised population

Table S2 – Adjusted vaccine effectiveness against COVID-19 outcomes in the immunocompromised population by vaccine types and periods of different dominant variant in the UK

Table S3 – Demographic characteristics of the immunocompromised population according COVID-19 outcomes (n=583,541)

*Figure S1 – Kaplan-Meier curves of first to fourth COVID-19 vaccine uptake in the immunocompromised and the general population*


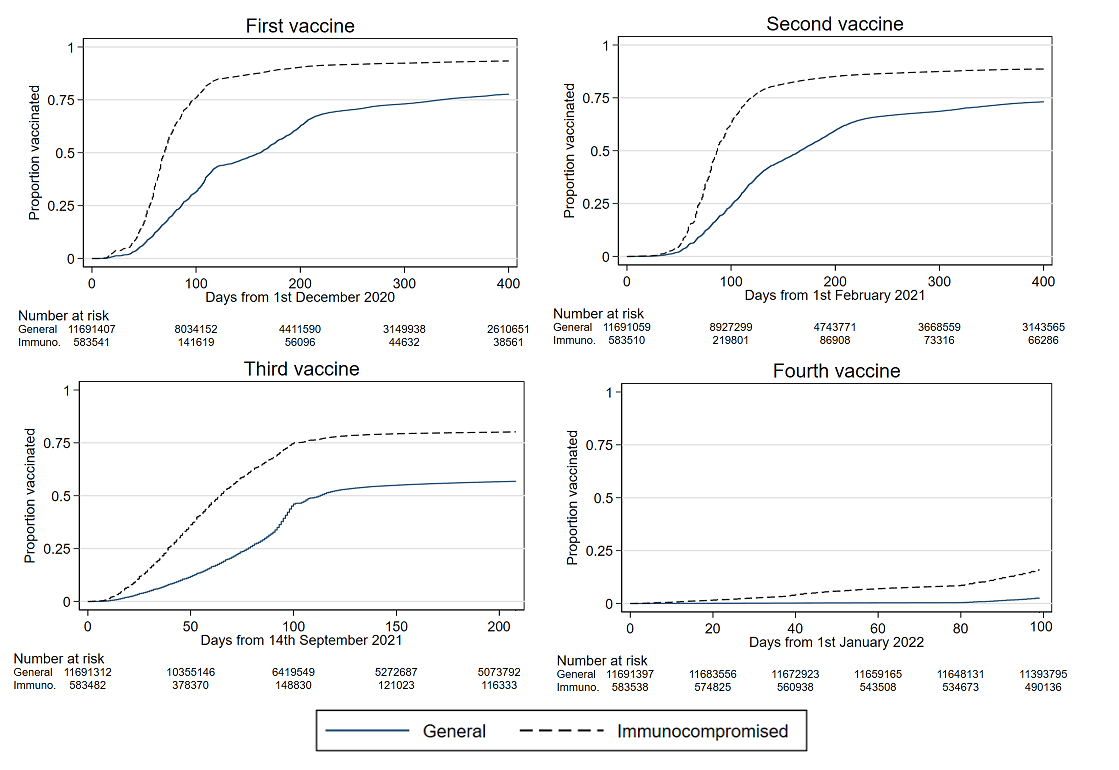


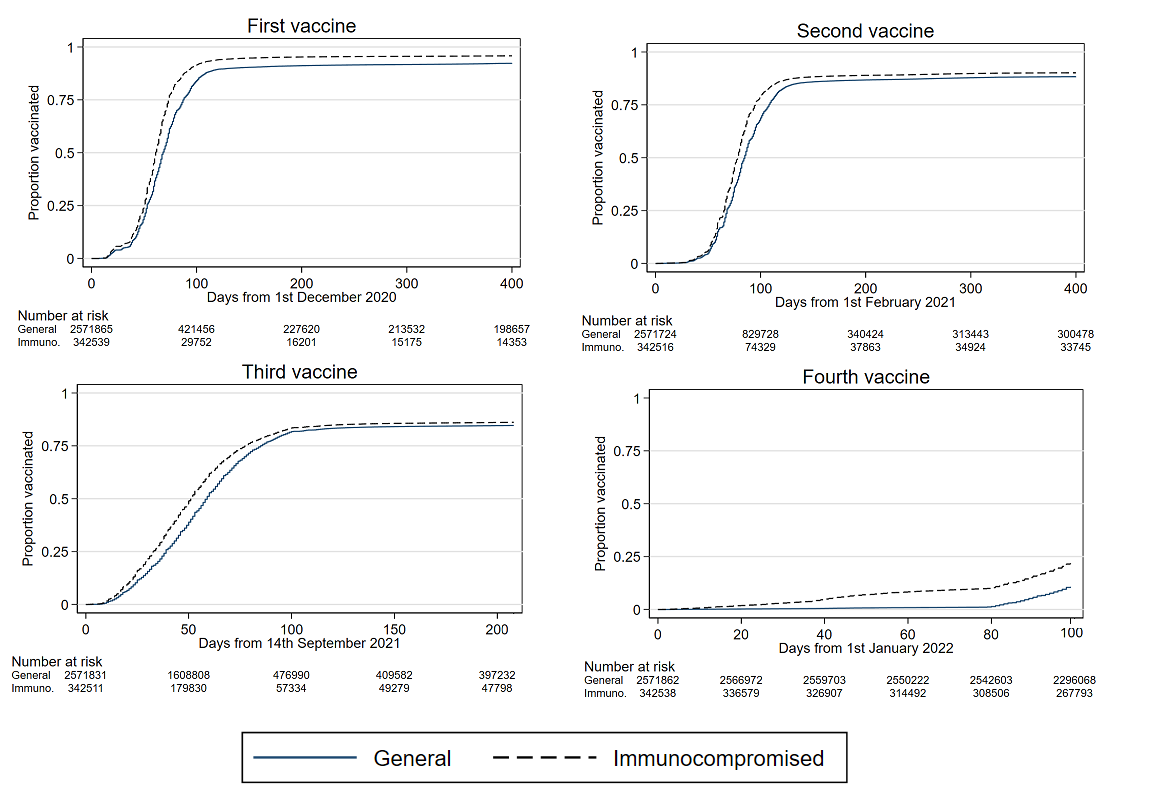
 *Figure S2 – Kaplan-Meier curves of first to fourth COVID-19 vaccine uptake in the immunocompromised and general population among people aged 60 and above.*

*Figure S3 –COVID-19 vaccine uptake by dose in the general and immunocompromised population*

**
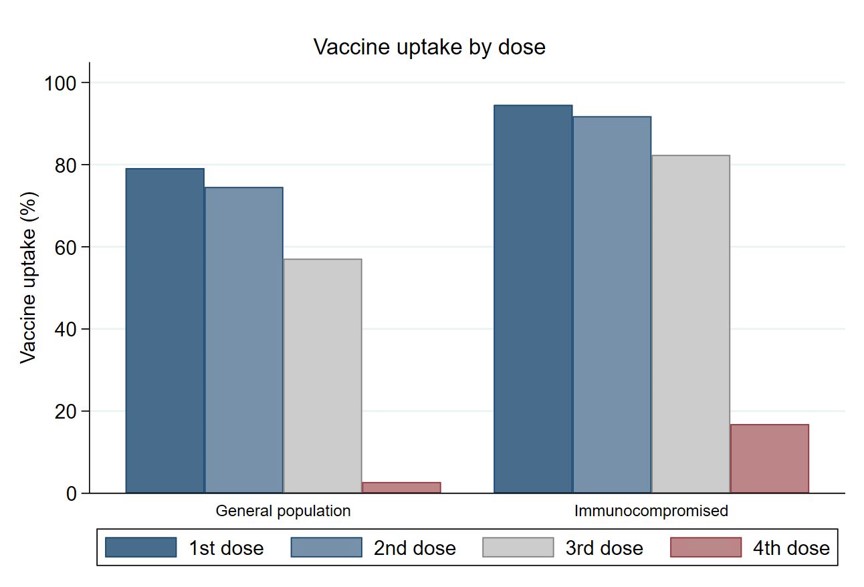
**

*Figure S4 – COVID-19 vaccine uptake by dose and vaccine types in the immunocompromised population*

**
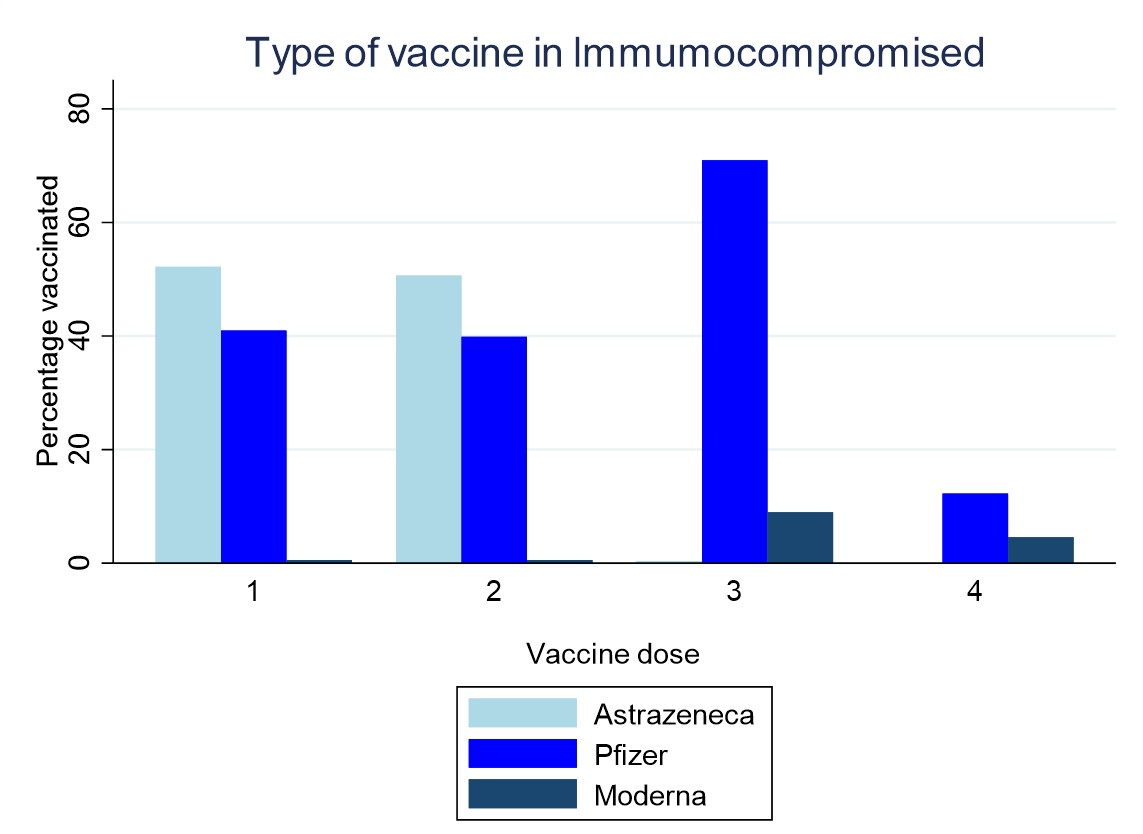
**

*Figure S5 – COVID-19 vaccine uptake by dose and subgroups in the immunocompromised population*

**
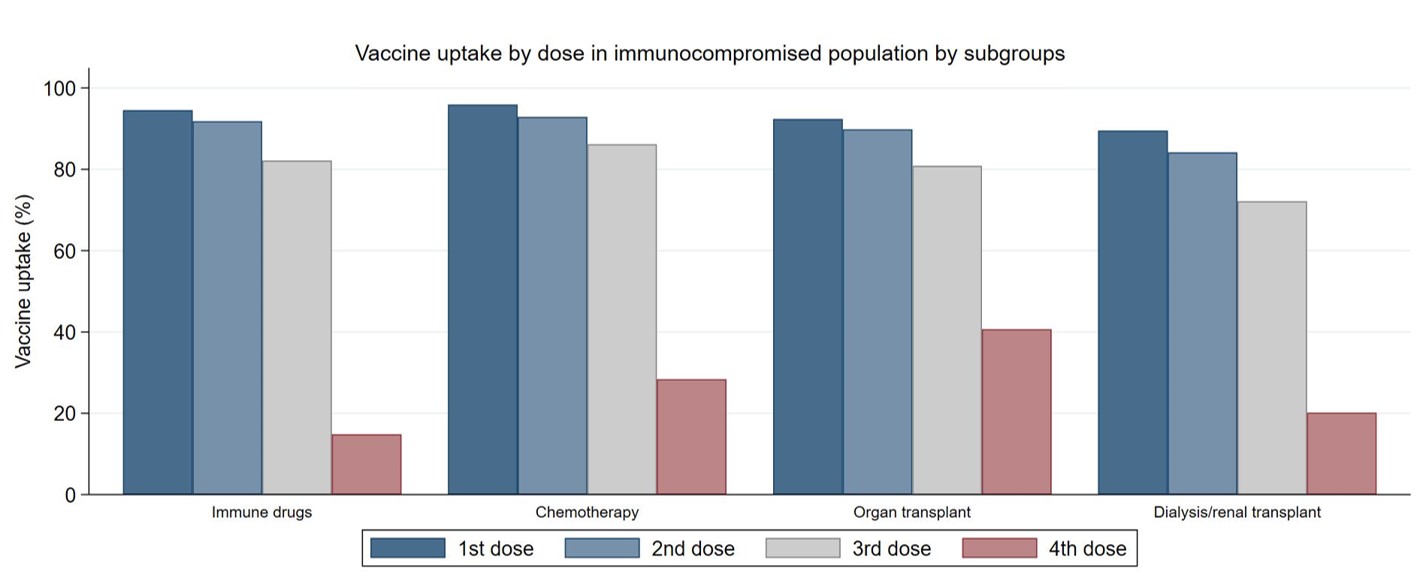
**

*Figure S6 – COVID-19 vaccine uptake by dose and ethnicity in the immunocompromised population*

**
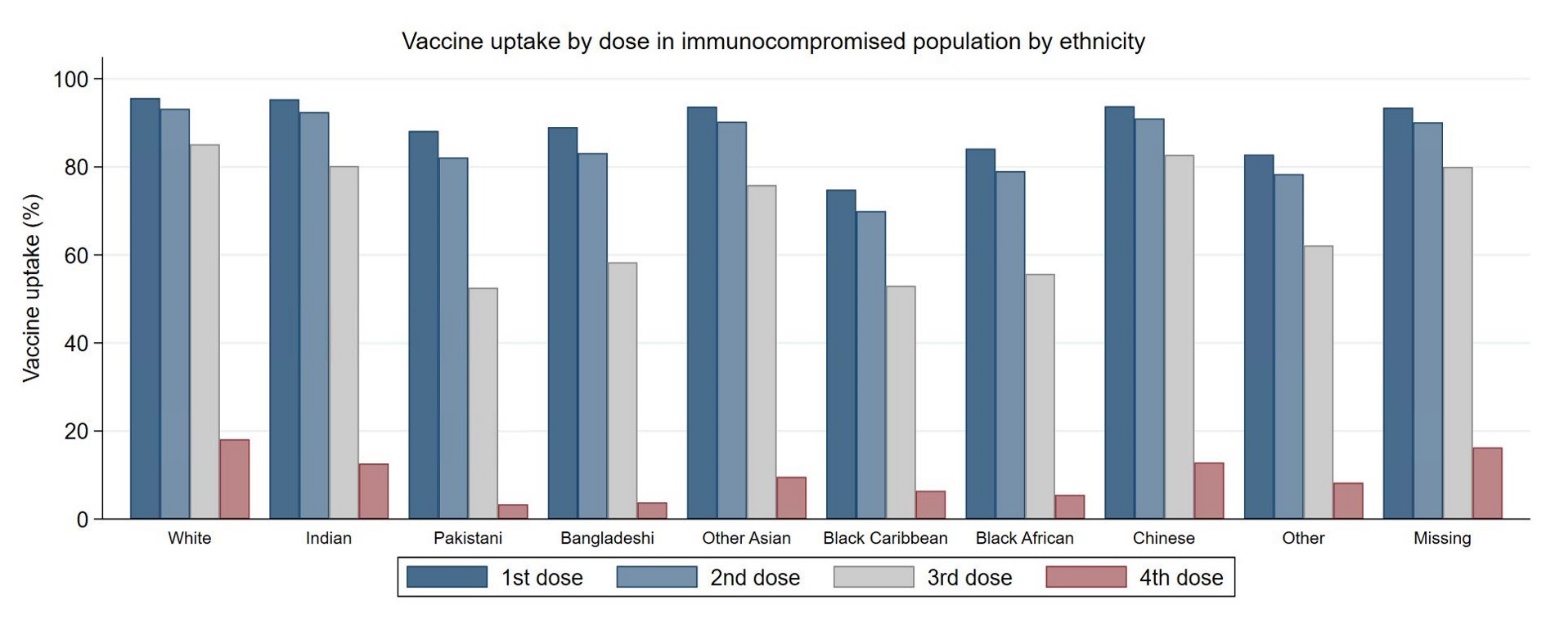
**

*Figure S7 – COVID-19 vaccine uptake by dose and deprivation quintile in the immunocompromised population*

**
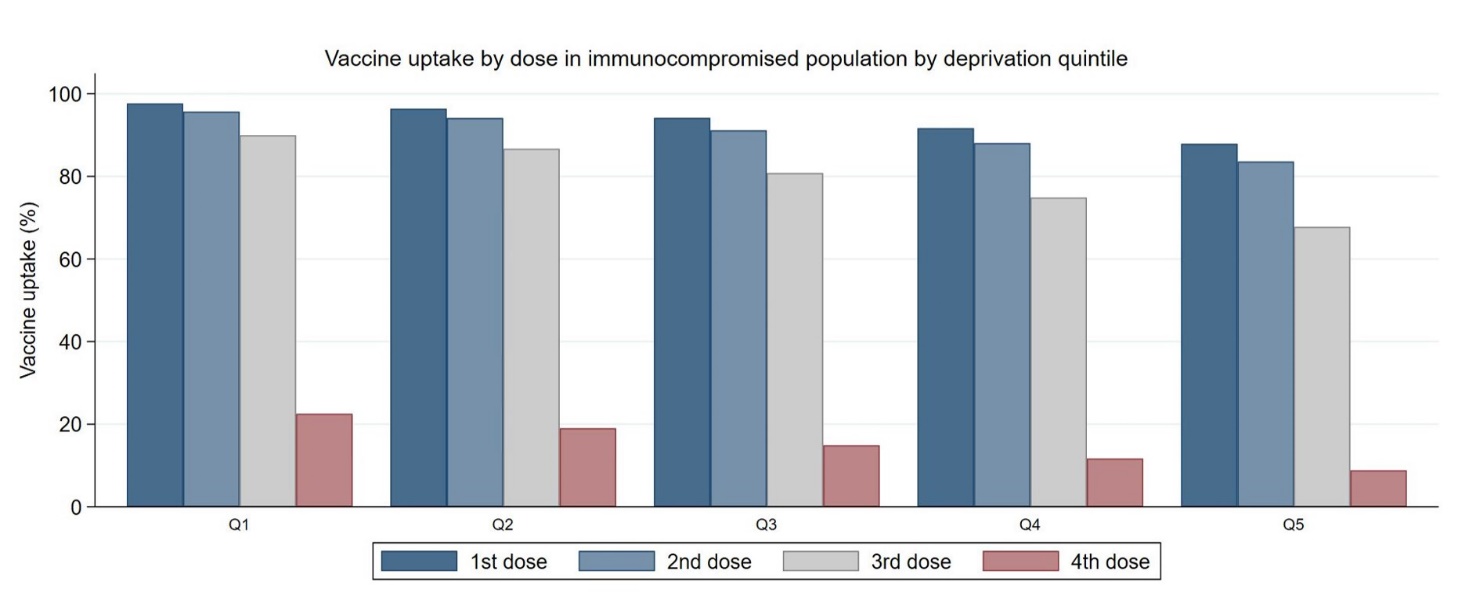
**

Note: Q1 to Q5 indicates quintiles of Townsend, from the most affluent (Q1) to the most deprived (Q5).

*Figure S8 – Adjusted vaccine effectiveness against COVID-19-realted hospitalisation by vaccine types in the immunocompromised population*


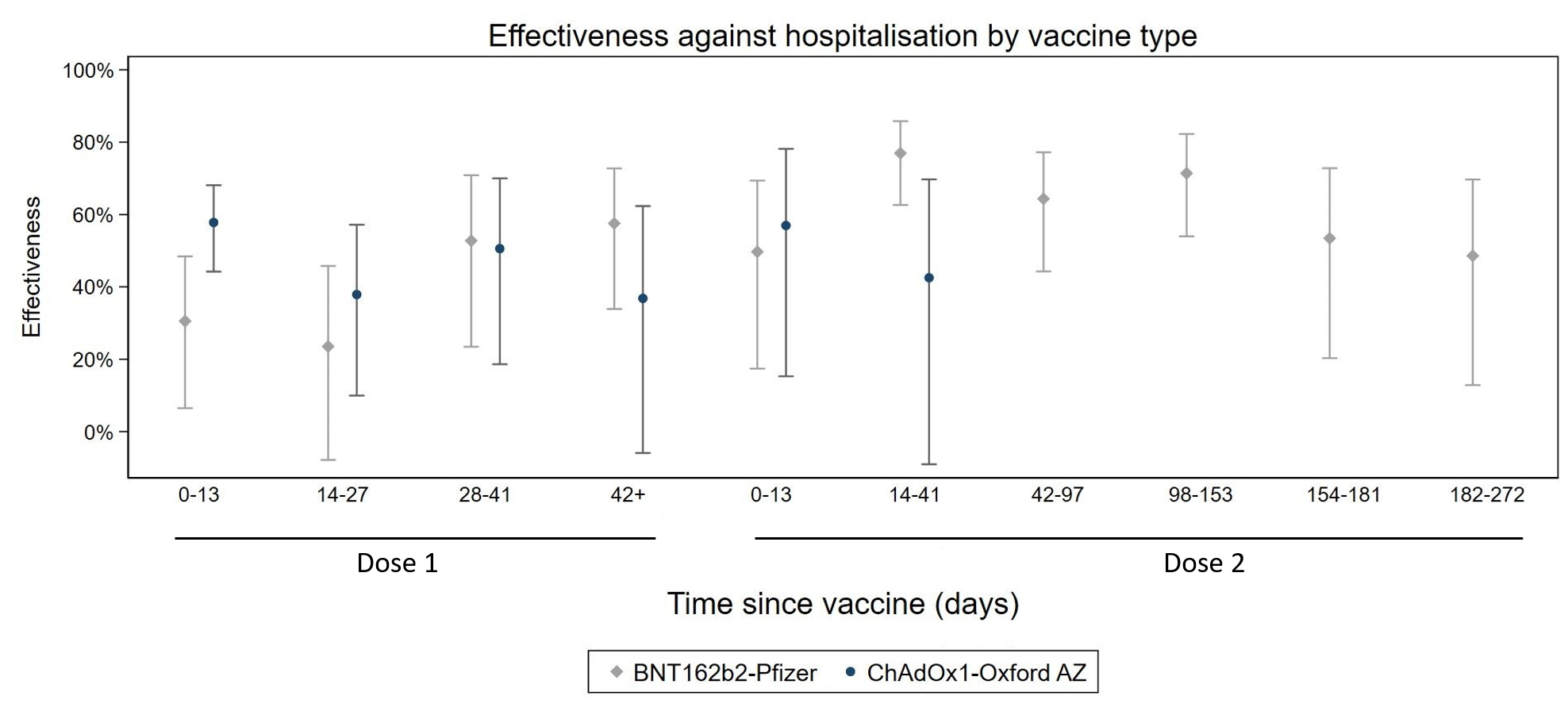


Note: Effectiveness by vaccine types was only estimated for risk against hospitalisation for ChAdOx1 (Oxford-AstraZeneca) and BNT162b2 (Pfizer) due to insufficient numbers or models not converging for other types of vaccine and outcomes; models were adjusted for ethnicity, Townsend, prior-COVID-19 infection, BMI, region, QCVOID comorbidities.

*Figure S9 – Adjusted vaccine effectiveness against COVID-19-realted hospitalisation in the immunocompromised population by periods of different dominant variant in the UK*


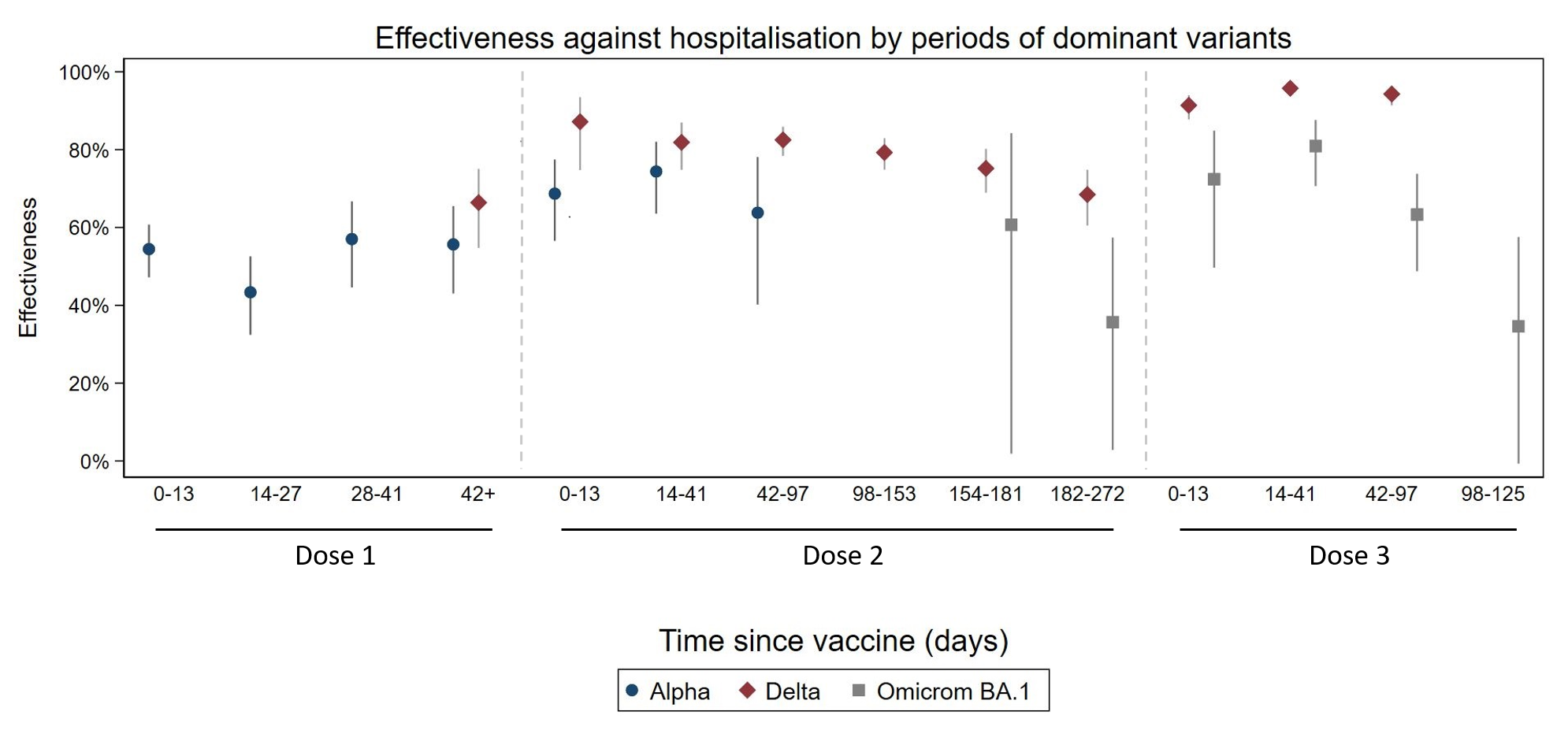


Note: Effectiveness by periods of different dominant variant in the UK was only estimated for risk against hospitalisation and due to insufficient numbers or models not converging in other outcomes; models were adjusted for ethnicity, Townsend, prior-COVID-19 infection, BMI, region, QCVOID comorbidities.

*Figure S10 – Adjusted vaccine effectiveness against COVID-19-realted death in the immunocompromised population by periods of different dominant variant in the UK*


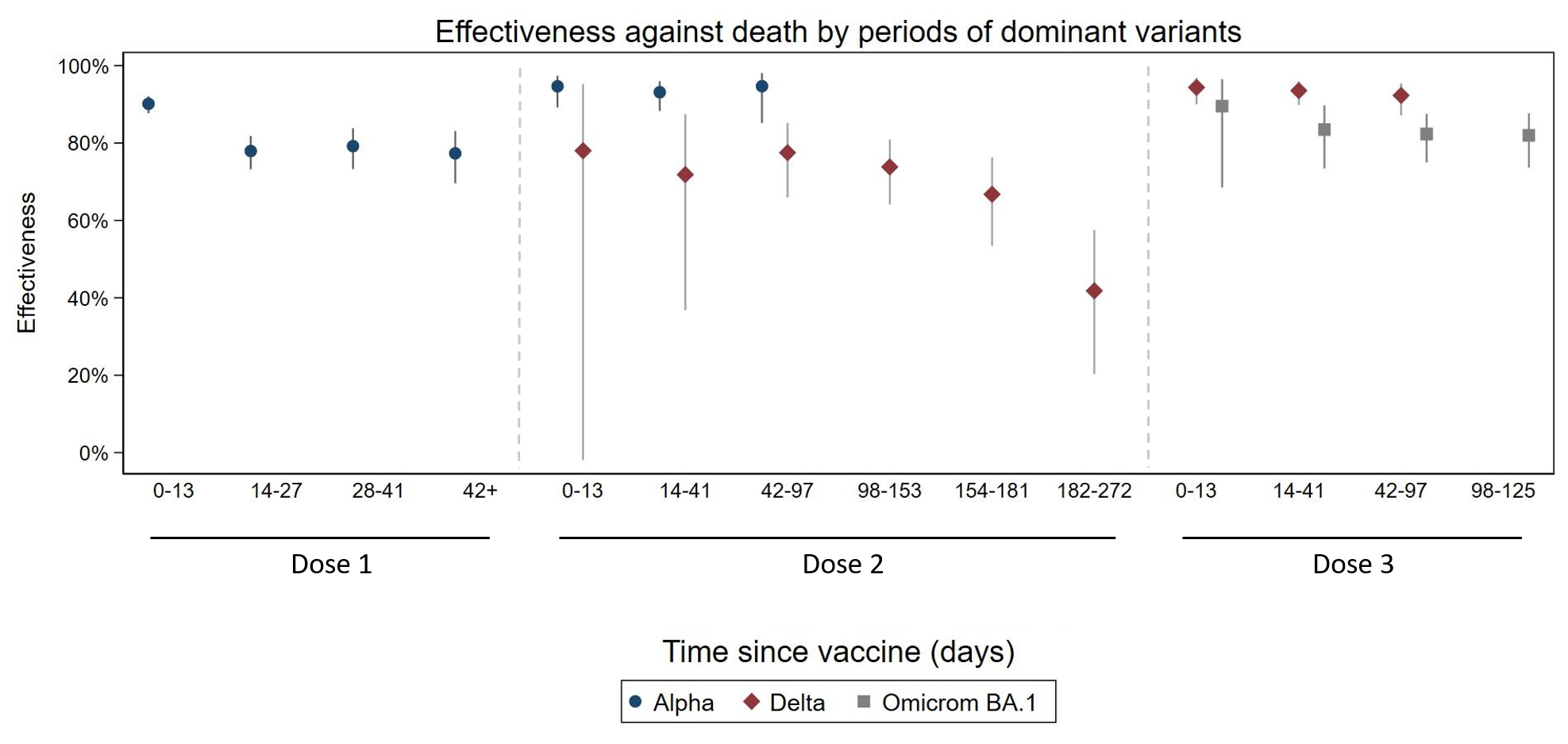


Note: Effectiveness by periods of different dominant variant in the UK was only estimated for risk against hospitalisation and due to insufficient numbers or models not converging in other outcomes; models were adjusted for ethnicity, Townsend, prior-COVID-19 infection, BMI, region, QCVOID comorbidities.

*
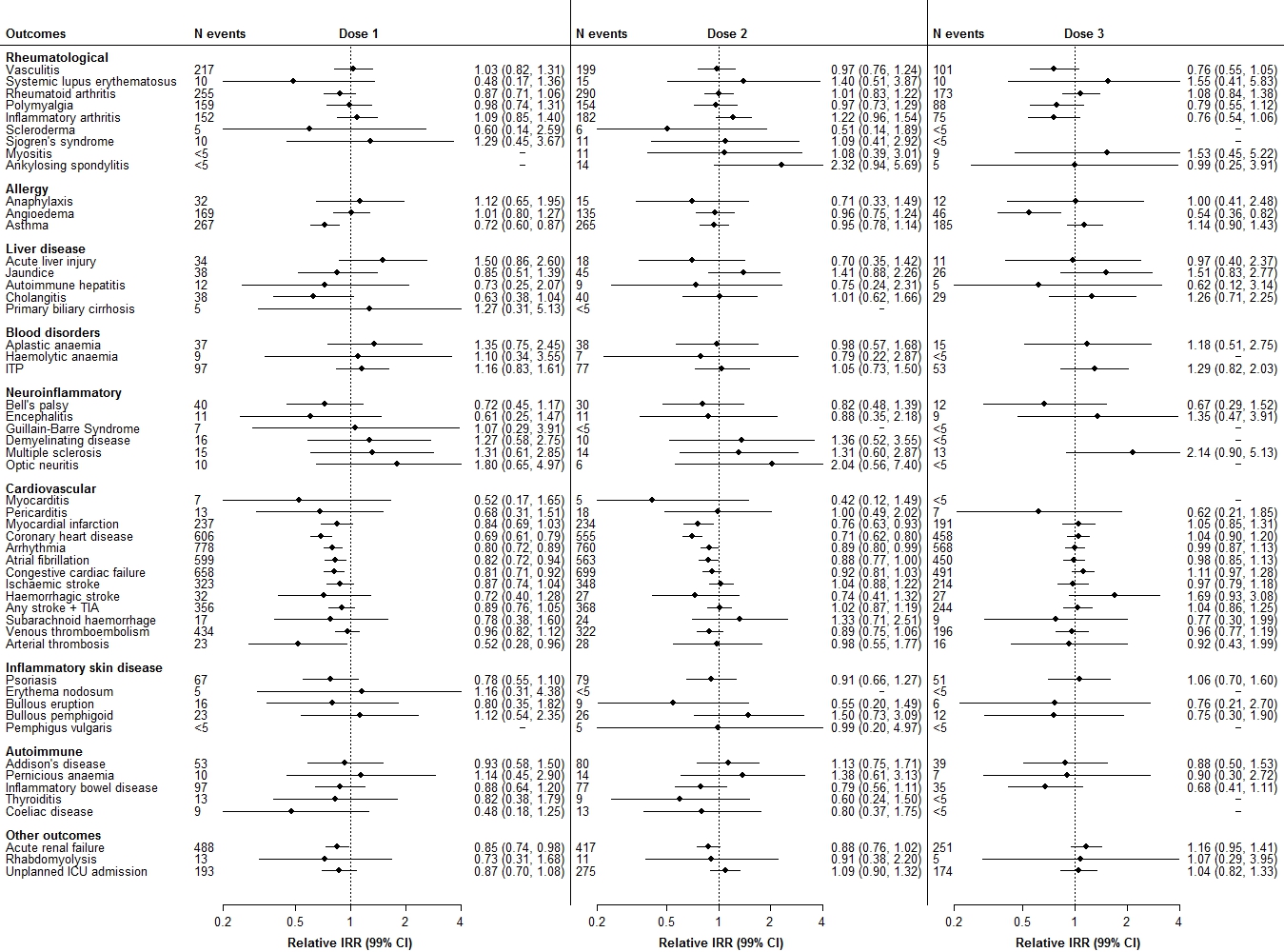
* *Figure S11 – Risk of serious outcomes following a first, second or third dose of COVID-19 vaccine in immunocompromised people relative to people who are not immunocompromised*

Note: N = number of events in the 1-28 days following vaccine dose in immunocompromised people; Relative incidence rate ratios (IRR 99% CI) in the 1-28 days following vaccination are presented for pre-specified outcomes where there were at least five events in the exposure period in immunocompromised people. Relative IRR of 1 indicates no difference in risk between immunocompromised people and non-immunocompromised people. ITP = idiopathic or immune thrombocytopenic purpura; TIA = transient ischaemic attack; ICU = intensive care unit.

*
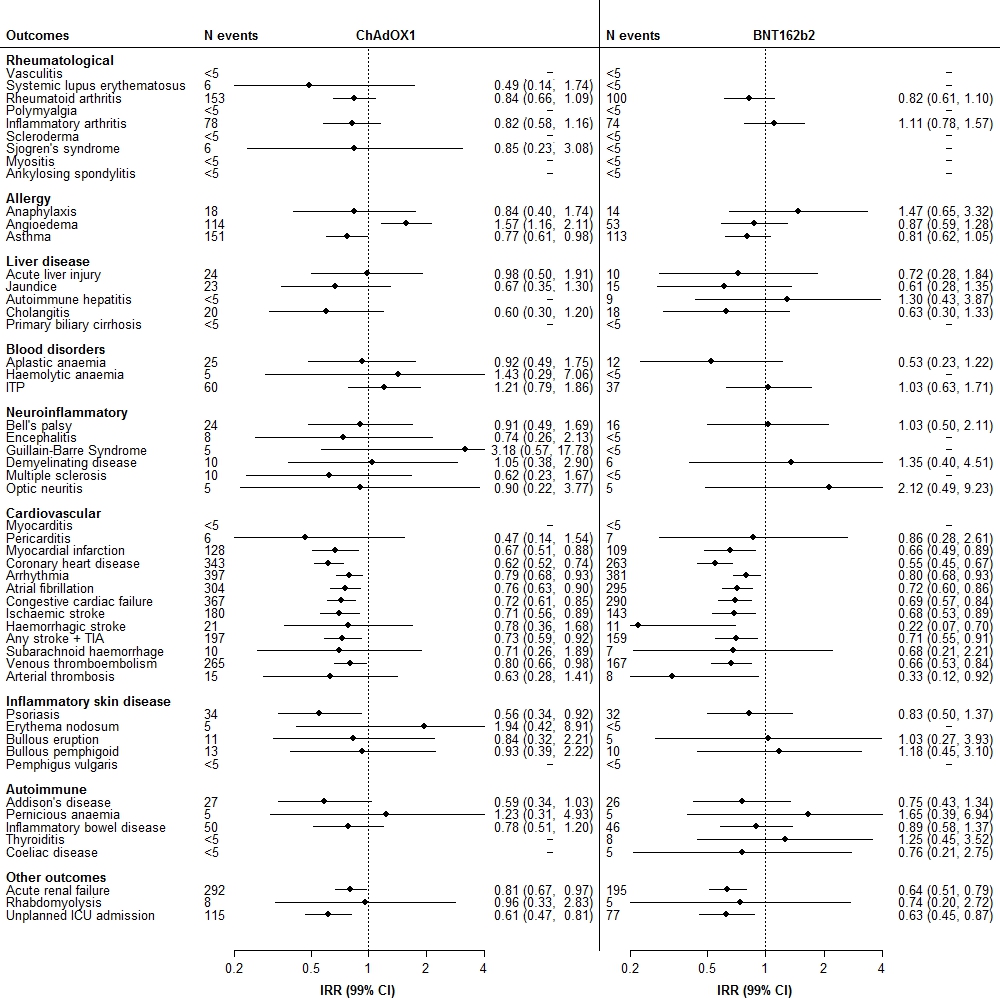
* *Figure S12 – Risk of serious outcomes following a first dose of ChAdOX1 or BNT162b2 vaccine in immunocompromised people*

Note: N = number of events in the 1-28 days following vaccine dose in immunocompromised people; incidence rate ratios (IRR 99% CI) in the 1-28 days following vaccination are presented for pre-specified outcomes only where there were at least five events following a first dose in immunocompromised people. ITP = idiopathic or immune thrombocytopenic purpura; TIA = transient ischaemic attack; ICU = intensive care unit.

*
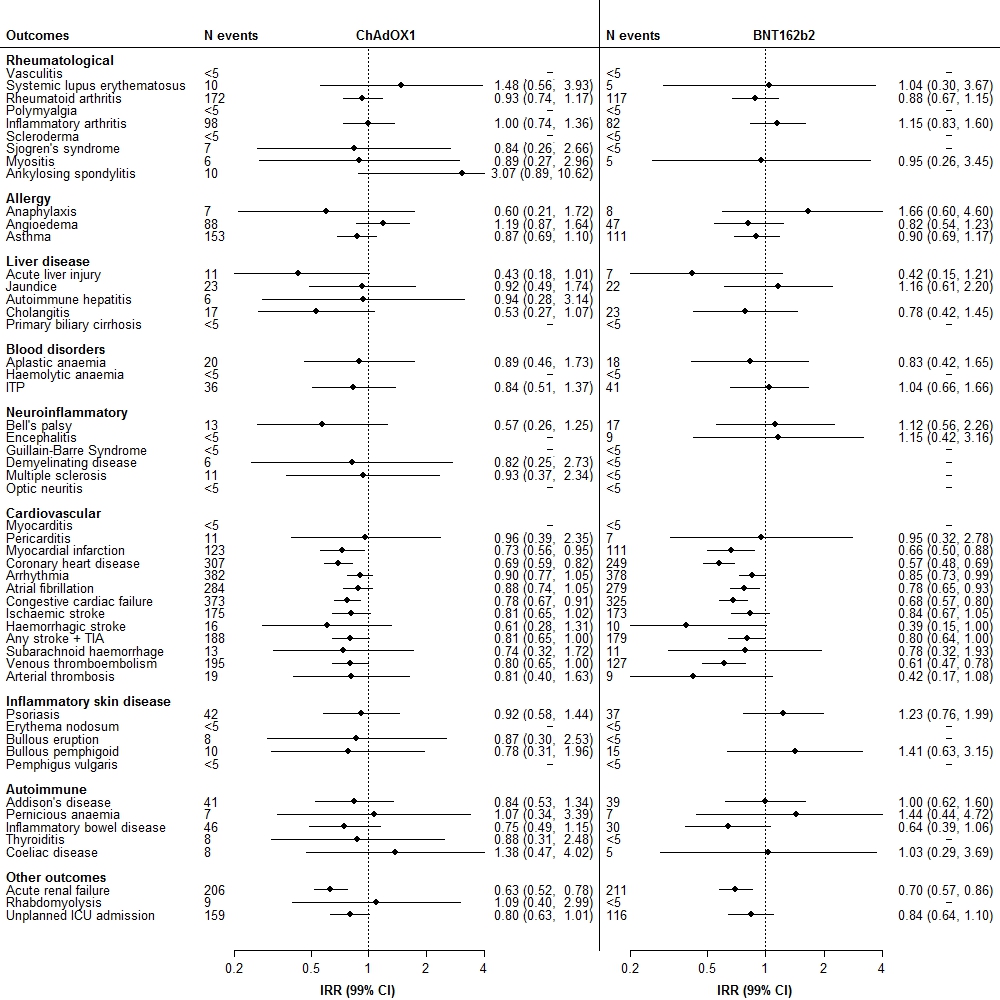
* *Figure S13 – Risk of serious outcomes following a second dose of ChAdOX1 or BNT162b2 vaccine in immunocompromised people*

Note: N = number of events in the 1-28 days following vaccine dose in immunocompromised people; incidence rate ratios (IRR 99% CI) in the 1-28 days following vaccination are presented for pre-specified outcomes only where there were at least five events following a third dose in immunocompromised people. ITP = idiopathic or immune thrombocytopenic purpura; TIA = transient ischaemic attack; ICU = intensive care unit.

*
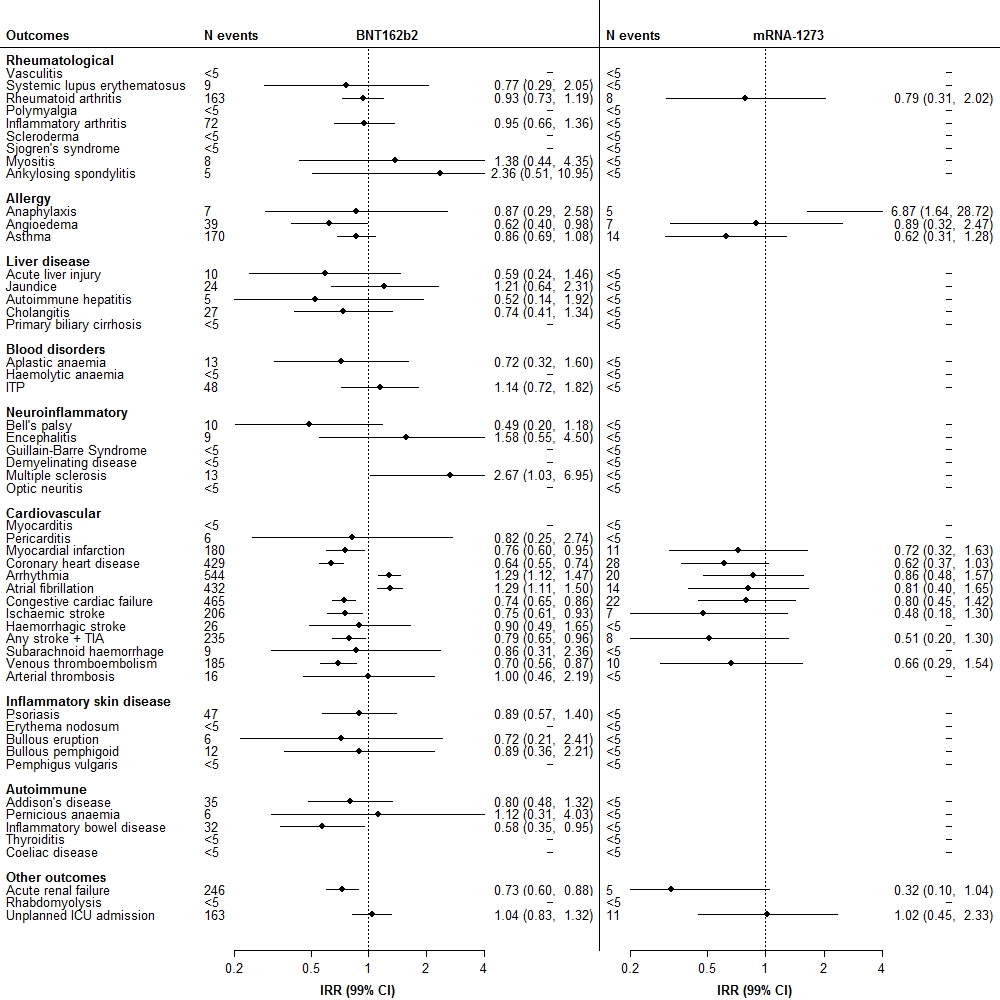
* *Figure S14 – Risk of serious outcomes following a third dose of BNT162b2 or mRNA-1273 vaccine in immunocompromised people*

Note: N = number of events in the 1-28 days following vaccine dose in immunocompromised people; incidence rate ratios (IRR 99% CI) in the 1-28 days following vaccination are presented for pre-specified outcomes only where there were at least five events following a third dose in immunocompromised people. ITP = idiopathic or immune thrombocytopenic purpura; TIA = transient ischaemic attack; ICU = intensive care unit.

*Table S1 –* *Multivariable Cox regression analyses of COVID-19 vaccine uptake in the immunocompromised population*

|  | **Cox proportional hazards with 95% CI of vaccination across all doses** | | | |
| --- | --- | --- | --- | --- |
|  | **Dose 1** | **Dose 2** | **Dose 3** | **Dose 4** |
| Age | 1.03 (1.03 - 1.03) | 1.02 (1.02 - 1.02) | 1.02 (1.02 - 1.02) | 1 (1 - 1) |
| Sex (ref. Male) | 0.94 (0.94 - 0.95) | 0.96 (0.95 - 0.96) | 1.01 (1 - 1.01) | 0.98 (0.97 - 0.99) |
| BMI (ref. Normal) |  |  |  |  |
| underweight | 0.85 (0.84 - 0.87) | 0.86 (0.84 - 0.87) | 0.78 (0.77 - 0.79) | 0.96 (0.94 - 0.97) |
| overweight | 1.01 (1 - 1.02) | 1.06 (1.05 - 1.07) | 1.09 (1.08 - 1.09) | 0.99 (0.98 - 0.99) |
| obese | 0.98 (0.97 - 0.99) | 1.07 (1.06 - 1.08) | 1.08 (1.07 - 1.09) | 0.96 (0.95 - 0.97) |
| severely obese | 0.98 (0.97 - 0.99) | 1.1 (1.09 - 1.11) | 1.08 (1.07 - 1.09) | 0.94 (0.94 - 0.95) |
| missing | 0.96 (0.95 - 0.97) | 0.98 (0.97 - 0.98) | 0.96 (0.95 - 0.97) | 0.96 (0.95 - 0.97) |
| Deprivation quintile  (ref. Q 1 [most affluent]) | | | | |
| Q 2 | 0.95 (0.94 - 0.96) | 0.95 (0.94 - 0.96) | 0.92 (0.91 - 0.93) | 0.97 (0.97 - 0.98) |
| Q 3 | 0.89 (0.89 - 0.9) | 0.9 (0.89 - 0.9) | 0.84 (0.83 - 0.84) | 0.95 (0.94 - 0.96) |
| Q 4 | 0.84 (0.83 - 0.84) | 0.85 (0.84 - 0.86) | 0.77 (0.76 - 0.77) | 0.93 (0.93 - 0.94) |
| Q 5 (most deprived) | 0.78 (0.77 - 0.79) | 0.8 (0.79 - 0.8) | 0.71 (0.71 - 0.72) | 0.92 (0.91 - 0.93) |
| Region of England  (ref. London) | | | | |
| East Midlands | 0.99 (0.97 - 1.01) | 0.99 (0.98 - 1.01) | 0.95 (0.93 - 0.97) | 0.99 (0.98 - 1.01) |
| East of England | 1 (0.98 - 1.01) | 1.05 (1.04 - 1.07) | 0.97 (0.96 - 0.99) | 0.99 (0.98 - 1.01) |
| North East | 0.99 (0.97 - 1.01) | 1.04 (1.03 - 1.06) | 0.97 (0.96 - 0.99) | 0.97 (0.96 - 0.99) |
| North West | 1 (0.99 - 1.01) | 1.03 (1.02 - 1.03) | 0.95 (0.94 - 0.96) | 0.99 (0.98 - 1) |
| South Central | 1.01 (1 - 1.03) | 1.03 (1.02 - 1.04) | 1 (0.99 - 1.01) | 1.01 (1 - 1.02) |
| South East | 0.95 (0.94 - 0.96) | 0.99 (0.98 - 1) | 0.94 (0.93 - 0.95) | 0.99 (0.98 - 1) |
| South West | 1.03 (1.02 - 1.04) | 1.01 (0.99 - 1.02) | 1.01 (1 - 1.02) | 1.04 (1.03 - 1.05) |
| West Midlands | 0.99 (0.98 - 1) | 1.02 (1.01 - 1.03) | 0.96 (0.95 - 0.97) | 0.99 (0.98 - 1) |
| Yorkshire & Humber | 1.01 (0.99 - 1.02) | 1.08 (1.07 - 1.1) | 1.01 (1 - 1.03) | 0.97 (0.96 - 0.99) |
| Ethnicity (ref. White) | | | | |
| Indian | 1.02 (1 - 1.04) | 1.01 (0.99 - 1.03) | 0.98 (0.96 - 1) | 0.96 (0.94 - 0.98) |
| Pakistani | 0.78 (0.76 - 0.79) | 0.78 (0.77 - 0.8) | 0.67 (0.65 - 0.68) | 0.92 (0.9 - 0.94) |
| Chinese | 0.94 (0.91 - 0.97) | 0.92 (0.89 - 0.94) | 0.82 (0.8 - 0.84) | 0.95 (0.92 - 0.97) |
| Bangladeshi | 0.97 (0.94 - 0.99) | 0.97 (0.95 - 1) | 0.93 (0.91 - 0.96) | 0.95 (0.93 - 0.97) |
| Other Asian | 0.55 (0.54 - 0.57) | 0.64 (0.63 - 0.66) | 0.62 (0.6 - 0.63) | 0.89 (0.87 - 0.92) |
| Black Caribbean | 0.75 (0.73 - 0.77) | 0.8 (0.78 - 0.83) | 0.72 (0.7 - 0.74) | 0.92 (0.9 - 0.95) |
| Black African | 0.87 (0.83 - 0.92) | 0.92 (0.87 - 0.97) | 0.98 (0.93 - 1.03) | 0.95 (0.9 - 1) |
| Other | 0.78 (0.77 - 0.8) | 0.82 (0.8 - 0.83) | 0.79 (0.78 - 0.81) | 0.95 (0.93 - 0.97) |
| Not recorded | 0.91 (0.91 - 0.92) | 0.92 (0.91 - 0.92) | 0.9 (0.9 - 0.91) | 0.98 (0.98 - 0.99) |

Note: Q1 to Q5 indicates quintiles of Townsend, from the most affluent (Q1) to the most deprived (Q5); Models were adjusted for QCVOID comorbidities.

*Table S2 – Adjusted vaccine effectiveness against COVID-19 outcomes in the immunocompromised population by vaccine types*

A.

|  | **COVID-19-related hospitalisation** | | | | | |
| --- | --- | --- | --- | --- | --- | --- |
|  | **ChAdOx1 (Oxford-AstraZeneca)** | | | **BNT162b2 (Pfizer)** | | |
|  | **VE** | **95%CI** | | **VE** | **95%CI** | |
| **No vaccine** | 0 | 0 | 0 | 0 | 0 | 0 |
| **1^st^ dose (days after)** | | | | | | |
| 0-13 | 0.58 | 0.44 | 0.68 | 0.31 | 0.06 | 0.48 |
| 14-27 | 0.38 | 0.1 | 0.57 | 0.24 | -0.08 | 0.46 |
| 28-41 | 0.51 | 0.19 | 0.7 | 0.53 | 0.23 | 0.71 |
| 42+ | 0.37 | -0.06 | 0.62 | 0.58 | 0.34 | 0.73 |
| **2^nd^ dose (days after)** | | | | | | |
| 0-13 days | 0.57 | 0.15 | 0.78 | 0.5 | 0.17 | 0.69 |
| 14-41 | 0.43 | -0.09 | 0.7 | 0.77 | 0.63 | 0.86 |
| 42-97 | 0.31 | -0.33 | 0.64 | 0.64 | 0.44 | 0.77 |
| 98-153 | ─ | ─ | ─ | 0.71 | 0.54 | 0.82 |
| 154-181 | ─ | ─ | ─ | 0.53 | 0.2 | 0.73 |
| 182-272 | ─ | ─ | ─ | 0.49 | 0.13 | 0.7 |

Note: Effectiveness by vaccine types was only estimated for risk against hospitalisation due to insufficient numbers or models not converging in other outcomes; models were adjusted for ethnicity, Townsend, prior-COVID-19 infection, BMI, region, QCVOID comorbidities; VE: vaccine effectiveness = (1-odds ratio)*100; ─: no data points due to insufficient numbers

B.

|  | **COVID-19-related hospitalisation** | | | | | | | | | **COVID-19-related death** | | | | | | | | | |
| --- | --- | --- | --- | --- | --- | --- | --- | --- | --- | --- | --- | --- | --- | --- | --- | --- | --- | --- | --- |
|  | **Alpha dominant** | | | **Delta dominant** | | | **Omicrom BA.1 dominant** | | | | **Alpha dominant** | | | **Delta dominant** | | | **Omicrom BA.1 dominant** | | |
|  | **VE** | **95%CI** | | **VE** | **95%CI** | | **VE** | **95%CI** | | | **VE** | **95%CI** | | **VE** | **95%CI** | | **VE** | **95%CI** | |
| **No vaccine** | 0 | 0 | 0 | 0 | 0 | 0 | 0 | 0 | 0 | | 0 | 0 | 0 | 0 | 0 | 0 | 0 | 0 | 0 |
| **1st dose (days after)** |  | | | | | | | | | | | | | | | | | | |
| 0-13 | 0.54 | 0.47 | 0.61 | ─ | ─ | ─ | ─ | ─ | ─ | | 0.9 | 0.88 | 0.92 | ─ | ─ | ─ | ─ | ─ | ─ |
| 14-27 | 0.43 | 0.32 | 0.53 | ─ | ─ | ─ | ─ | ─ | ─ | | 0.78 | 0.73 | 0.82 | ─ | ─ | ─ | ─ | ─ | ─ |
| 28-41 | 0.57 | 0.45 | 0.67 | ─ | ─ | ─ | ─ | ─ | ─ | | 0.79 | 0.73 | 0.84 | ─ | ─ | ─ | ─ | ─ | ─ |
| 42+ | 0.56 | 0.43 | 0.65 | 0.66 | 0.55 | 0.75 | ─ | ─ | ─ | | 0.77 | 0.7 | 0.83 | ─ | ─ | ─ | ─ | ─ | ─ |
| **2nd dose (days after)** |  | | | | | | | | | | | | | | | | | | |
| 0-13 days | 0.69 | 0.57 | 0.77 | 0.87 | 0.75 | 0.93 | ─ | ─ | ─ | | 0.95 | 0.89 | 0.97 | 0.78 | -0.02 | 0.95 | ─ | ─ | ─ |
| 14-41 | 0.74 | 0.64 | 0.82 | 0.82 | 0.75 | 0.87 | ─ | ─ | ─ | | 0.93 | 0.88 | 0.96 | 0.72 | 0.37 | 0.87 | ─ | ─ | ─ |
| 42-97 | 0.64 | 0.4 | 0.78 | 0.83 | 0.78 | 0.86 | ─ | ─ | ─ | | 0.95 | 0.85 | 0.98 | 0.78 | 0.66 | 0.85 | ─ | ─ | ─ |
| 98-153 | ─ | ─ | ─ | 0.79 | 0.75 | 0.83 | ─ | ─ | ─ | | ─ | ─ | ─ | 0.74 | 0.64 | 0.81 | ─ | ─ | ─ |
| 154-181 | ─ | ─ | ─ | 0.75 | 0.69 | 0.8 | 0.36 | 0.03 | 0.57 | | ─ | ─ | ─ | 0.67 | 0.53 | 0.76 | ─ | ─ | ─ |
| 182-272 | ─ | ─ | ─ | 0.68 | 0.61 | 0.75 | 0.41 | -0.23 | 0.72 | | ─ | ─ | ─ | 0.42 | 0.2 | 0.58 | ─ | ─ | ─ |
| **3^rd^ dose (days after)** |  | | | | | | | | | | | | | | | | | | |
| 0-13 days | ─ | ─ | ─ | 0.91 | 0.88 | 0.94 | 0.72 | 0.5 | 0.85 | | ─ | ─ | ─ | 0.94 | 0.9 | 0.97 | 0.9 | 0.68 | 0.97 |
| 14-41 | ─ | ─ | ─ | 0.96 | 0.94 | 0.97 | 0.81 | 0.71 | 0.88 | | ─ | ─ | ─ | 0.94 | 0.9 | 0.96 | 0.83 | 0.73 | 0.9 |
| 42-97 | ─ | ─ | ─ | 0.94 | 0.91 | 0.96 | 0.63 | 0.49 | 0.74 | | ─ | ─ | ─ | 0.92 | 0.87 | 0.95 | 0.82 | 0.75 | 0.88 |
| 98-125 | ─ | ─ | ─ | ─ | ─ | ─ | 0.35 | -0.01 | 0.58 | | ─ | ─ | ─ | ─ | ─ | ─ | 0.82 | 0.74 | 0.88 |
| 126-153 | ─ | ─ | ─ | ─ | ─ | ─ | ─ | ─ | ─ | | ─ | ─ | ─ | ─ | ─ | ─ | 0.81 | 0.71 | 0.87 |

Note: Effectiveness by periods of different dominant variant in the UK was only estimated for risk against hospitalisation and death due to insufficient numbers or models not converging in other outcomes; models were adjusted for ethnicity, Townsend, prior-COVID-19 infection, BMI, region, QCVOID comorbidities; VE: vaccine effectiveness = (1-odds ratio)*100; ─: no data points due to insufficient number

*Table S3 – Demographic characteristics of the immunocompromised population according COVID-19 outcomes (n=583,541)*

|  | **COVID-19 outcomes** | | |
| --- | --- | --- | --- |
|  | **Hospitalisations** | **Death** | **ICU admission** |
| Total, N (%) | 17,817 (3.1) | 5,478 (0.9) | 1,249 (0.2) |
| Immunocompromised conditions (%) | | | |
| Transplant | 811 (4.6) | 193 (3.5) | 78 (6.2) |
| Dialysis or renal transplant | 1108 (6.2) | 298 (5.4) | 131 (10.5) |
| Immune-modifying drugs | 15964 (89.6) | 4860 (88.7) | 1096 (87.8) |
| Chemotherapy | 1757 (9.9) | 749 (13.7) | 63 (5.0) |
| Age, years mean (SD) | 67.8 (16.1) | 76.9 (12.0) | 60.8 (13.2) |
| Female sex (%) | 8590 (48.2) | 2390 (43.6) | 494 (39.6) |
| BMI, kg/m^2^ mean(SD) | 29.5 (6.7) | 28.0 (6.5) | 31.6 (6.8) |
| Townsend quintile of deprivation (%) | | | |
| Q 1 (most affluent) | 3840 (21.6) | 1388 (25.3) | 230 (18.4) |
| Q 2 | 3792 (21.3) | 1262 (23.0) | 233 (18.7) |
| Q 3 | 3678 (20.6) | 1122 (20.5) | 249 (19.9) |
| Q 4 | 3501 (19.6) | 1005 (18.3) | 268 (21.5) |
| Q 5 (most deprived) | 2894 (16.2) | 681 (12.4) | 263 (21.1) |
| Ethnicity (%) | | | |
| White | 12913 (72.5) | 4004 (73.1) | 781 (62.5) |
| Indian | 486 (2.7) | 113 (2.1) | 52 (4.2) |
| Pakistani | 523 (2.9) | 113 (2.1) | 51 (4.1) |
| Bangladeshi | 257 (1.4) | 61 (1.1) | 33 (2.6) |
| Other Asian | 273 (1.5) | 50 (0.9) | 47 (3.8) |
| Black Caribbean | 323 (1.8) | 86 (1.6) | 32 (2.6) |
| Black African | 342 (1.9) | 67 (1.2) | 50 (4.0) |
| Chinese | 38 (0.2) | 10 (0.2) | 4 (0.3) |
| Other | 461 (2.6) | 93 (1.7) | 54 (4.3) |
| Not recorded | 2201 (12.4) | 881 (16.1) | 145 (11.6) |
